# Podocytes Produce and Secrete Functional Complement C3 and Complement Factor H

**DOI:** 10.3389/fimmu.2020.01833

**Published:** 2020-08-14

**Authors:** Anne K. Mühlig, Lindsay S. Keir, Jana C. Abt, Hannah S. Heidelbach, Rachel Horton, Gavin I. Welsh, Catherine Meyer-Schwesinger, Christoph Licht, Richard J. Coward, Lars Fester, Moin A. Saleem, Jun Oh

**Affiliations:** ^1^University Children's Research@Kinder-UKE, University Medical Center Hamburg-Eppendorf, Hamburg, Germany; ^2^Department of Pediatric Nephrology, University Children's Hospital, University Medical Center Hamburg-Eppendorf, Hamburg, Germany; ^3^Bristol Renal and Children's Renal Unit, University of Bristol, Bristol, United Kingdom; ^4^Center of Experimental Medicine, Institute of Cellular and Integrative Physiology, University Medical Center Hamburg-Eppendorf, Hamburg, Germany; ^5^Division of Pediatric Nephrology, The Hospital for Sick Children, Toronto, ON, Canada; ^6^Department of Neuroanatomy, University Medical Center Hamburg-Eppendorf, Hamburg, Germany; ^7^Institute for Anatomy and Cell Biology, Friedrich-Alexander-Universität Erlangen-Nürnberg, Erlangen, Germany

**Keywords:** complement secretion, proteinuria, glomerulus, kidney, local regulation

## Abstract

Podocytes are an important part of the glomerular filtration barrier and the key player in the development of proteinuria, which is an early feature of complement mediated renal diseases. Complement factors are mainly liver-born and present in circulation. Nevertheless, there is a growing body of evidence for additional sites of complement protein synthesis, including various cell types in the kidney. We hypothesized that podocytes are able to produce complement components and contribute to the local balance of complement activation and regulation. To investigate the relevant balance between inhibiting and activating sides, our studies focused on complement factor H (CFH), an important complement regulator, and on C3, the early key component for complement activation. We characterized human cultured podocytes for the expression and secretion of activating and regulating complement factors, and analyzed the secretion pathway and functional activity. We studied glomerular CFH and C3 expression in puromycin aminonucleoside (PAN) -treated rats, a model for proteinuria, and the physiological mRNA-expression of both factors in murine kidneys. We found, that C3 and CFH were expressed in cultured podocytes and expression levels differed from those in cultivated glomerular endothelial cells. The process of secretion in podocytes was stimulated with interferon gamma and located in the Golgi apparatus. Cultured podocytes could initiate the complement cascade by the splitting of C3, which can be shown by the generation of C3a, a functional C3 split product. C3 contributed to external complement activation. Podocyte-secreted CFH, in conjunction with factor I, was able to split C3b. Podocytes derived from a patient with a CFH mutation displayed impaired cell surface complement regulation. CFH and C3 were synthesized in podocytes of healthy C57Bl/6-mice and were upregulated in podocytes of PAN treated rats. These data show that podocytes produce functionally active complement components, and could therefore influence the local glomerular complement activation and regulation. This modulating effect should therefore be considered in all diseases where glomerular complement activation occurs. Furthermore, our data indicate a potential novel role of podocytes in the innate immune system.

## Introduction

Proteinuric kidney diseases are very common in adult and pediatric patients (1–3). In these diseases the glomeruli of the kidneys are damaged and patients then suffer from severe proteinuria. Podocytes (glomerular epithelial cells) are a critical part of the glomerular filtration barrier, which also consists of the glomerular basement membrane (GBM) and endothelial cells. They show a complex, highly developed architecture and one of their main tasks is to maintain the structure of the glomerular filter. Structurally, podocytes form major processes and smaller foot processes. The foot processes of neighboring podocytes interdigitate with each other and form the slit-diaphragm, which works as a semi-selective membrane and maintains the filtration of urine. Thus, larger plasma proteins, such as albumin, are held back in the serum. Podocytes also secrete proteins such as VEGF, which not only maintains their own phenotype but also maintains the fenestrated glomerular endothelium (4). When podocytes are damaged, their foot processes are effaced and the filter becomes leaky and patients then experience proteinuria. Proteinuria also occurs in complement-associated renal diseases (5, 6).

The complement system is an important player in the innate immune system. It consists of more than 30 proteins (7). C3 activation is a central step in the initiation and amplification of the system. All pathways result in the formation of a C3 convertase, which hydrolyses C3 to generate C3a and C3b. C3b, together with factor B form another C3 convertase (C3bBb) and together with another C3b fragment a C5 convertase (C3b2Bb), which leads to the formation of the terminal complement complex (TCC/C5b-9) (7). The C5b-9 complex can lead to destruction of invading pathogens, foreign or apoptotic cells or, in sublytic concentrations, to an activation of several cell types. Furthermore, the occurring split products (e.g., C3a and C5a) act as anaphylatoxins and recruit additional immune cells. Beyond the long-known function of complement components in the initiation of the complement cascade—especially C3—seem to play a role in differentiation, proliferation and immune response in T-cells and therefore link the innate to the adaptive immunity (8).

Serum-based and membrane-bound complement inhibitors regulate the activation of the complement system, to prevent inappropriate activation. Complement factor H (CFH) is one of the most important circulating regulators of the alternative pathway. It serves as an essential cofactor for complement factor I (CFI)-mediated C3b cleavage and has important tasks in the decay of C3 convertase (9, 10). Therefore, C3 and CFH can be hypothesized to be important protagonists in the initiation and regulation of the complement cascade.

There is uncontrolled complement activation in a number of renal diseases, notably atypical hemolytic uremic syndrome (aHUS) (11), C3 glomerulopathy (10, 12, 13), IgA nephropathy (14), and membranous nephropathy glomerulopathy (10, 12, 13), IgA nephropathy (14), and membranous nephropathy (6). The activation of the complement system is thought to cause the system or an uncontrolled regulation. Besides these diseases, which are directly triggered by the systemic complement cascade, the complement system can also be activated locally in non-primary immune-mediated diseases, for example in Shiga toxin-associated hemolytic uremic syndrome or kidney transplantation (15). This secondary toxin-associated hemolytic uremic syndrome or kidney transplantation (15). This aggravate severe renal damage. The reason the glomerulus is particularly vulnerable to unregulated systemic or local complement activation is not fully clarified, yet.

Most complement proteins are produced in the liver and circulate in the blood. In recent years, several extrahepatic sources have been reported, including the kidney, brain, lungs, and intestines. Several studies have shown these organs can also produce small amounts of complement proteins, which could contribute to local complement activation and regulation (16–20). The kidney is one of the most important extrahepatic sources. It has been shown that kidney-derived C3 contributes up to 10% of the circulating C3 (21). The local production of complement proteins in the human kidney has already been described in healthy individuals (22) and in those with glomerular diseases (23–27). Primary renal cell culture studies confirmed that mesangial, endothelial, and epithelial cells are capable of synthesizing the activating circulating complement proteins C3, C4, and factor B (28–32). CFH mRNA was also detected in human glomeruli B (28–32). CFH mRNA was also detected in human glomeruli (33). Sheerin and co-workers demonstrated that local complement production is and that the loss of local complement synthesis in this proteinuria model prevents severe renal damage (34). Based on these findings, a recently published study demonstrates that podocytes, key players in the development of proteinuria, may themselves be one important source of complement C3 in proteinuric diseases (35). Furthermore, Zoshima and colleagues suggested a potential role of podocyte derived CFH in clearing immune complexes in a murine model of immune-complex glomerulonephritis (36). However, the production of complement proteins by podocytes remains controversial.

Podocytes do not have any direct contact to serum-based complement activation, which is in most cases the reason for complement-mediated renal damage. Nevertheless, in special circumstances the glomerular filtration barrier is leaky and allows components of the blood to pass through, thus permitting contact with podocytes. Additionally, local complement activation could contribute to podocyte damage. Furthermore, there is a growing body of evidence that podocytes may act as immune modulating cells (37, 38). It has been shown that podocytes express several toll-like receptors (TLR) (39–42), which are regularly expressed in macrophages, T- and B-cells and B7-1, which is expressed by B-cells or antigen-presenting cells. Even though the role of TLR and B7-1 in podocytes is highly debated, these data indicate that podocytes may have additional roles beyond the stabilization of the glomerular filtration barrier.

Therefore, we hypothesized that podocytes produce functionally active complement proteins. While most studies have focused either on the activating complement perspective or the expression of regulating factors, we were interested in both aspects to give a more complete overview. We investigated the expression, functionality and secretion mechanisms of the activating central complement factor C3 and the regulating CFH in human podocyte cells and in a rat model of proteinuria.

## Materials and Methods

### Antibodies, Proteins, and Serum

For Western blot antibodies against human C3 (goat, Pierce, Thermo Scientific, Waltham, MA, USA, catalog number: PA1-29715), CFH (goat, Calbiochem, Merck, Darmstadt, Germany, catalog number: 341276), CD 46 (LS Bio, Seattle, WA, USA, catalog number: 6754), C5b-9 (abcam, Cambridge, UK, rabbit catalog number: ab55811), which seems to recognize a neo-epitope on C9 and was used in the assay with rat-podocytes, C5b-9 (mouse, Pierce, catalog number: MA5-28502) were used in dilution 1:500–1,000. Mitogen-activated protein kinases (MAPK) and β-actin were evaluated with rabbit antibodies from Cell Signaling (1:100–500, Cell Signaling Technology, Danvers, MA, USA, catalog numbers: 4668, 9101, 4631, 9252, 9102, 9212). For immunofluorescence antibodies against C3 (goat, Pierce, 1:100), CFH (goat, Calbiochem, 1:100), giantin (rabbit, 1:100, abcam, catalog number: ab24586), tubulin and laminin (both rabbit, 1:100, Sigma, St. Louis, MO, USA, catalog numbers: T3526, L9393) were used. Nuclei were stained with 4, 6-Diamidine-2-phenylindole dihydrochloride (DAPI) (1:10,000, Sigma, St. Louis, MO, USA). IRDye conjugated secondary antibodies for Western blot were obtained from Licor (1:5,000, Lincoln, NE, USA). Appropriate secondary antibodies for immunofluorescence were used from Thermo Fisher (1:200–400, Waltham, MA, USA): Alexa Fluor, goat anti-rabbit A488 and donkey anti-goat A594. Normal human serum (NHS) was obtained from healthy laboratory staff (10 persons, pooled and stored in aliquots at −20°C). Recombinant CFH, CFI, C3 and C3b were purchased from Merck (Darmstadt, Germany).

### Animal Experiments

Animals were housed in a controlled animal facility with free access to water and standard animal chow. Experiments were performed according to national and institutional animal care and ethical guidelines and have been approved by the local ethical committees at the Medical University of Hamburg-Eppendorf and the Ministry of Health and Consumer Protection. Puromycin aminonucleoside-induced nephropathy (PAN) was induced in male Sprague–Dawley rats by intraperitoneal injection of puromycin aminonucleoside in phosphate buffered saline (PBS) (Sigma) as described (43). Urine was collected in metabolic cages and proteinuria was measured in semi-quantitative conventional dip sticks (Siemens, Berlin, Germany). Rats were euthanized on day 28 and glomeruli were isolated: kidneys were encapsulated, cortex was minced and sieved through a 90 μm filter (BD, Franklin Lakes, NJ, USA). The filtrate was sieved through a 53 μm filter (BD) and glomeruli were removed from this filter with cold PBS. Glomeruli were centrifuged at 1,000 rpm and washed three times with 0.5% BSA in PBS (Sigma). Glomeruli were frozen and kept at −80°C until mRNA isolation. Small parts of the kidney cortex were fixed in formaldehyde and paraffin embedded for light microscopy. Sections 1–2 μm were stained with periodic acid-Schiff reagent (Sigma) according to the manufacturer's instructions.

### Cell Culture

Primary human podocytes were transfected with temperature sensitive SV40 and cultured as previously described (44). These cells were used for all experiments with human podocytes. The phenotype-differentiation of the cells was routinely controlled in the microscope, and differentiation markers were analyzed repeatedly as cited in the original manuscript (44). Cells were used below passage 25. RPMI-media with glutamine was supplemented with 10% fetal bovine serum (FBS) (both Gibco, Life Technologies, Carlsbad, CA, USA) 100 U/ml penicillin, 0.1 mg/ml streptomycin (Gibco), and insulin, transferrin, and sodium selenite mixture (ITS) (Sigma). Conditionally immortalized human glomerular endothelial cells (CiGenC) were produced from primary glomerular endothelial cells in a similar way (45). Cells were used till passage 30, and provided with the microvascular endothelial cell growth media EGM2^TM^-MV (Lonza, Basel, Switzerland). Podocytes from an aHUS patient with a known Arg1182Ser (G3546T) CFH mutation were isolated from a nephrectomized kidney with informed consent. This mutation affects the ability of CFH to bind to C3b and heparin (46). Cells were isolated as described in (44) and transfected with SV40.

To test protein secretion, differentiated podocytes were washed with PBS, incubated in serum-free RPMI-media (SFM) for 12–36 h and supernatant samples were taken. Human interferon γ (IFNg) (R&D Systems, Wiesbaden, Germany) was used with final concentrations from 0.1 to 100 ng/ml. Brefeldin A (BFA) (Sigma), diluted in ethanol, was used at 5 ng/ml and applied for 0–8 h.

Primary rat podocytes were cultivated from rats on embryonic day 18. Kidneys were removed and washed twice with Hank's Balanced Salt Solution (HBSS) (Gibco). Papain replaced HBSS for enzymatic tissue digestion. After 30 min of shaking at 37°C, digestion was stopped by three RPMI washes. Kidneys were homogenized and passed through a 40 μm cell strainer. Cells were seeded at 20,000 cells/ml in RPMI 1640 medium, containing 10% FBS, penicillin-streptomycin 100 U/ml, sodium pyruvate and HEPES (all Gibco) at 37°C. Differentiation occurred after 2 weeks.

### Quantitative PCR (qPCR) and Conventional PCR

RNA was then isolated using an innuPrep RNA Mini Kit (Analytik Jena, Biometra, Jena, Germany). RNA concentration was measured with a NanoDrop ND-1000 spectrophotometer (Thermo Scientific) (47). Two micrograms of RNA was converted to cDNA using a cDNA synthesis kit (Life Technologies, Carlsbad, CA, USA). qPCR was performed using SYBR green (Sigma), according to manufacturer's instructions on the StepOnePlus real-time PCR system (Applied Biosystems, Life Technologies) (95°C 20 s, 60°C 20 s, 72°C 20 s). Sequences were obtained from the Harvard Primer bank (http://pga.mgh.harvard.edu/primerbank/) and purchased from Life Technologies: for human CFH forward: CCTGATCGCAAGAAAGACCAG and reverse: ACTGAACGGAATTAGGTCCAAAT and for C3 forward: CCAAGAGCTCAAGGTGAGGG and reverse: GGAACGGACAACGAGGACTT. The primer sequences for rat CFH forward TTAGGCTGGCAGTTGGATCT, CFH reverse TCCACCCATCTGCATCACAT C3 forward TCAGGGTCCCAGCTACTAGT, and C3 reverse AGTCTCTTCACTCTCCAGCC. Relative expression was determined as compared to 18S controls.

In conventional (reverse transcriptase) PCR the following primers from Life Technologies were used for C3: GCTGCTCCTGCTACTAACCCA (forward), AAAGGCAGTTCCCTCCACTTT (reverse), and for CFH: ACATTACTTCATTCCCGTTGTC (forward), ATACTCCAGTTTCCCATCCCAA (reverse). PCR was accomplished using dNTPs (Life Technologies), Dream Taq Polymerase-set (Fermentas, Thermo Scientific), and Biometra Thermocycler (Labrepco, Horsham, PA, USA) (94°C 2 min; 94°C 1 min, 60°C 1 min, 72°C 1 min x 34; 72°C 2 min). PCR-product was loaded onto a 1.5% agarose gel with a 100 bp DNA Ladder (Biomol, Hamburg, Germany). Additional primer sequences are shown in Supplementary Table 1.

### Immunofluorescence

Cells were grown on coverslips and fixed in 4% paraformaldehyde (PFA) (Sigma), as previously described (48). Cells were blocked with 3% bovine serum albumin, and permeabilized with 0.05% Triton-X 100 (both Sigma), when needed. Images were taken using the Leica confocal imaging spectrophotometer system (TCS-SP2) or Leica TCS SP5 (Leica, Wetzlar, Germany). Imaging was done with HCX PL FLUOTAR L 40x 0.6 objective lens with an optical zoom of 40, or with 63x HCX PL APO Lbd. Bl. Oil. Microscopes were used at the Wolfson Imaging Facility, University of Bristol or at the University Microscope Imaging Facility (UMIF), University Medical Center Hamburg-Eppendorf.

### Western Blotting

Lysates were made with RIPA lysis buffer (Sigma), gel electrophoresed (4–12% Gels, Invitrogen, Carlsbad, CA, USA). Samples were boiled with 4 × NuPAGE LDS Sample Buffer (Invitrogen) and 0.1 M dithiothreitol and blotted onto PVDF (Merck Millipore) membranes as previously described (47, 48). Membranes were blocked with Odyssey^®^ Blocking Buffer (Licor), 60 min at room temperature prior to overnight primary antibody incubation at 4°C. Seeblue protein standard (Invitrogen) was used as marker. Blots were washed and incubated with appropriate secondary antibody before being imaged using an Odyssey^®^ Clx Blot Scanner for IRDye-antibodies. Densitometry was performed using Quantity One software (v4.6.5, BioRad Laboratories, Hercules, CA, USA).

### Cofactor Assay

Cofactor activity of secreted CFH was tested as previously described (49, 50). In short, together with recombinant C3b (final concentration 6 μg/ml) and recombinant CFI (final concentration 40 μg/ml) podocyte supernatant (48 h in SFM) was incubated at 37°C. Samples were taken from the reaction after 0, 5, and 20 min. The addition of reduced SDS sample buffer (Invitrogen) and boiling the samples for 10 min stopped the reaction. The samples were then separated by SDS page, as described for Western blotting. C3 split products were detected with anti C3 antibody. Recombinant CFH (final concentration 33 μg/ml) was used in the assay for positive control.

### Complement Challenge Assays

Human podocytes were washed in PBS prior to treatment with 30% rabbit serum in gelatin veronal buffer (GVB) (Sigma) to set up antigen antibody complexes on the cell surface. Complement was then activated by adding 20% CFH-deficient human serum (Complement Technology, Tyler, TX, USA) in GVB for 30 min (51). Complement activation was then detected by Western blot against C5b-9.

Rat podocytes were serum starved for 24 h and incubated with anti-Fx1A-IgG (0.5 mg/ml), produced as previously described (52). Briefly, sheep were immunized with isolated brush border proteins, and serum was taken. IgG was isolated with Nab^TM^ Spin Kit for antibody purification (Thermo Scientific). After incubation for 20 min at room temperature, 2.5% NHS, or 2.5% C3-depleted human serum (Calbiochem, Merck Millipore), was added for 40 min at 37°C. Cells were harvested for Western blotting.

### C3 Convertase Assay

The C3 convertase (MicroVue, Quidel, San Diego, CA, USA) assay was used following the manufacturer's instructions. C3a was measured at 450 nm using ultra-microplate reader EL808 (BioTek) and KC Junior software (v.1.41.6). A ratio of sample and untreated control was calculated. Supernatants were taken from plates with defined cell number and measured within 2 h.

### Factor H Re-synthesis Assay

Human podocytes were grown for immunofluorescence and treated with trypsin (Gibco) 10 μg/ml for 15 min at 37°C (53). Cells were then incubated with SFM for 24 h.

### Fluorescence *in situ* Hybridization

Kidneys from wildtype untreated C57BL/6 mice were dissected. Kidneys were fixed in RNase free 4% PFA overnight at 4°C and cryopreserved in 30% sucrose, cryo-sectioned (14 μm) and stored frozen at −20°C until use. The Affymetrix Quantigene View RNA (Affymetrix, Santa Clara, CA, USA) *in situ* hybridization system was used as per manufacturer's instructions. Sections were thawed and dried at 60°C prior to Protease Q (20 min, 40°C) treatment. Probes for CFH (accession No: NM_009888, Catalog No VB1-16095) and C3 (accession No: NM_009778, Catalog No VB1-13781), purchased from Affymetrix, were applied at 40°C for 4 h. A no probe control was run alongside each experiment. The probe was labeled using fast red dye (Affymetrix). After washing, slides were blocked in DAKO blocking reagent (Dako, Hamburg, Germany). Rabbit anti-laminin antibody was diluted in antibody diluting reagent (Dako) and incubated overnight at 4°C. Secondary antibody (1:200) diluted in antibody diluting reagent, was added after washing, and incubated for 3 h. DAPI nuclear counter stain was applied prior to mounting using Fluoromount and then imaged using Leica SP5.

### Statistical Analysis

Statistical analyses and graphs were carried out using PRISM (Version 5, GraphPad Software). Results were considered significant when *p* < 0.05. Images were analyzed with ImageJ.

## Results

### Human Podocytes Express and Secrete Complement Factors C3 and CFH *in vitro*

**Figure 1:**
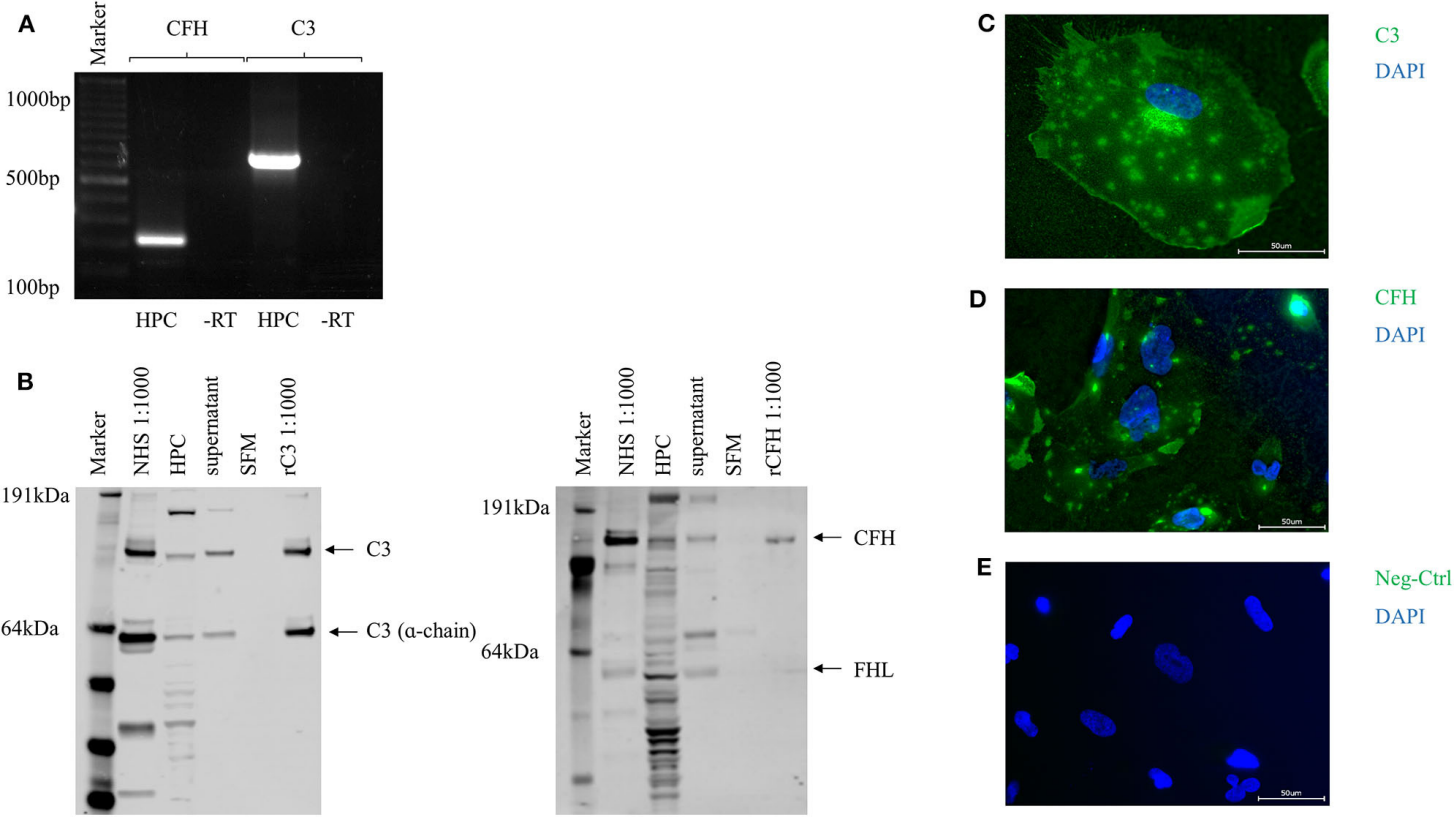
Complement proteins C3 and CFH were expressed and secreted by unstimulated human podocytes (HPC). **(A)** Detection of PCR products for C3 (320 bp) and CFH (783 bp) in conventional reverse transcriptase PCR compared to -RT control (-RT, control of cDNA without addition of RT; bp, basepairs). **(B)** Protein expression for C3 and CFH was determined by Western blot from whole cell podocyte lysate (HPC) and cell culture supernatant after 24 h in serum-free media (SFM). Normal human serum (NHS, 1:1,000) and recombinant CFH or C3 were used as positive controls (rC3/rCFH, 1:1,000), SFM was used as negative control. In the C3 Western blot also the α-chain of C3 can be detected. The CFH gene has two protein products: CFH and factor H like-protein (FHL). Both are detected in this Western blot in the podocyte cell lysate, in NHS and to a small extend in the cell culture supernatant (representative Western blots, *n* = 4). **(C)** Protein expression of C3 and **(D)** CFH was confirmed in immunofluorescence on the surface of non-permeabilized cultured podocytes compared to isotype negative control (Neg-Ctrl) **(E)**. (*n* = 3, C3 and CFH = green, nucleus = blue, scale bar 50 μm, 40x).

To evaluate the potential of podocytes to build complement components we analyzed conditionally immortalized human podocytes. Conventional reverse transcriptase PCR identified mRNA for the activating key component C3 (PCR product 783 base-pairs [bp]) and also for CFH (320 bp) (Figure 1A). RNA was also identified for the early activating complement proteins C1q, C1r, C1s, C2, C4, C5; the alternative pathway activators factor B, factor D, properdin and the regulators CD55, CD59, and CD46 (MCP) (Supplementary Figure 1). As CFH is the most important soluble inhibitor of the alternative pathway, and C3 is a key component in early complement activation, our subsequent work focused specifically on these two proteins. After 24 h of incubation in SFM, C3 and CFH were detected with Western blots of the whole cell lysates and in the cell culture-supernatant. Podocytes showed the expression of two products of the C3 and the CFH-gene, which probably corresponds to the α-chain of C3 and factor H-like protein (FHL) (Figure 1B). Complement proteins for C2, C5, and the regulatory components CFH, CD46, CD55, and CD59 were also detected (data not shown). The expression of C3 and CFH could also be determined in immunofluorescence (Figures 1C–E). Both proteins were detected on the surface of non-permeabilized podocytes.

### Production and Secretion of Podocyte Complement Components Is an Active Process

**Figure 2:**
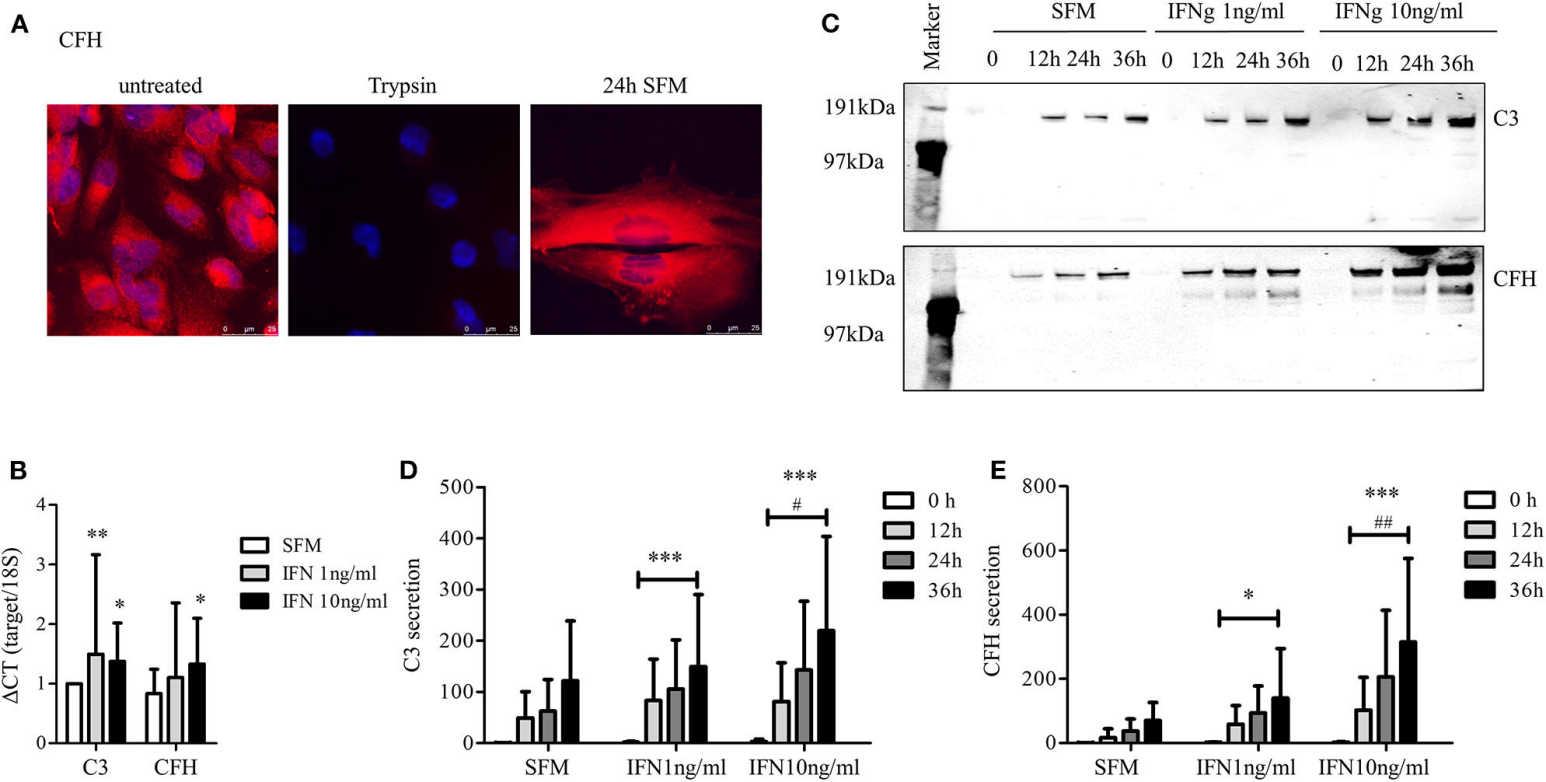
Production and secretion of C3 and CFH is an active and inducible process. **(A)** CFH was removed from the surface of cultured podocytes using low dose trypsin (10 μg/ml, 3 min), the cells were then incubated in SFM for 24 h and the expression of CFH on the cell surface is determined in immunofluorescence staining. The pictures are showing the detection of CFH (in red) before treatment with trypsin (left), immediately after the treatment (middle) and after 24 h recovery in SFM (right) (*n* = 3, DAPI = blue, 60x, scale bar 25 μm). **(B–E)** Expression and secretion of C3 and CFH was enhanced by interferon γ (IFNg). **(B)** mRNA expression of C3 and CFH in human podocytes incubated 6 h with SFM (white bars), IFNg 1 ng/ml (gray bars), and IFNg 10 ng/ml (black bars) (*p < 0.05, **p < 0.001 compared to expression in SFM). **(C)** Western blot against C3 (above) and CFH (below) of supernatant from podocytes incubated in SFM, IFNg 1 ng/ml or IFNg 10 ng/ml for 0–36 h. Quantitative analysis of Western blots for the secretion of C3 **(D)** and CFH **(E)** from podocytes incubated in SFM, IFNg 1 and 10 ng/ml for 0 (white bars), 12 (light gray bars), 24 (dark gray), and 36 (black bars) hours (*p < 0.05 and ***p < 0.001 compared to secretion with SFM, ^#^*p* < 0.05 and ^##^*p* < 0.01 compared to incubation with IFNg 1 ng/ml) Data are shown in mean and SD. Mann Whitney *U*-test, *n* = 5 independent experiments in **(B,D,E)**.

Secreted CFH circulates throughout the body and can bind to most cells by binding to the cellular glycocalyx. This regulates uncontrolled complement activation directly on the cell surface. CFH glycocalyx binding sites can be degraded temporarily by treatment with low dose trypsin (54). To see whether the podocytes are capable of replacing removed CFH from the surface we treated differentiated podocytes with low dose trypsin to remove surface-bound CFH. Cells were then allowed to recover in SFM. CFH was detected again on the surface of the podocytes 24 h later, showing that these cells can produce and replace CFH (Figure 2A).

IFNg has previously been shown to increase cellular synthesis of complement proteins in a variety of cell lines (55–57). Therefore, human podocytes were treated with IFNg at different time points and concentrations. Stimulation with IFNg significantly enhanced human podocyte mRNA expression of C3 and CFH (Figure 2B). It also increased the expression of both proteins in whole cell lysates (data not shown). Furthermore, secreted C3 and CFH were increased after stimulation with IFNg in a time- and dose-dependent manner (Figures 2C–E). This suggests that podocytes are capable of producing complement proteins as part of a pro-inflammatory response.

### Expression of Complement Factor C3 and CFH Varies in Cultured Human Podocytes and Glomerular Endothelial Cells

**Figure 3:**
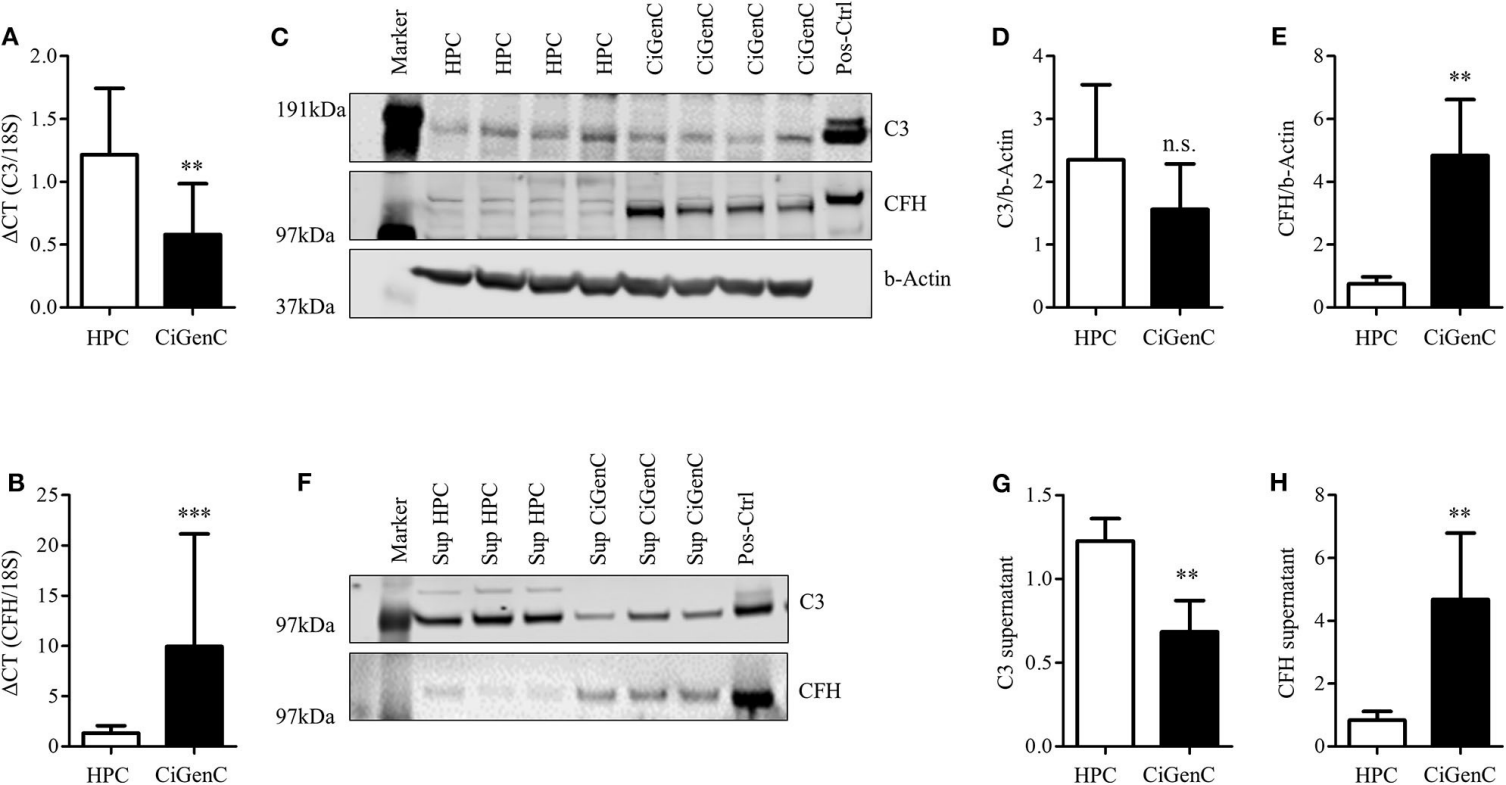
Expression of complement factor C3 and CFH is different in cultured podocytes and glomerular endothelial cells. Conditionally immortalized human podocytes (HPC) and human glomerular endothelial cells (CiGenC) were compared for their expression of C3 and CFH on mRNA- and protein-level and secretion of both proteins. **(A)** mRNA-expression of C3/18S and **(B)** CFH/18S in HPC (white bars) and CiGenC (black bars) (*n* = 12). Protein expression of C3 and CFH was analyzed in Western blot **(C)** of whole cell lysates. Densitometry of C3 **(D)** and CFH **(E)** in HPC (white bars) and CiGenC (black bars) normalized to expression of beta (b)-actin (*n* = 6). Secretion of C3 and CFH into cell culture supernatant was tested in similar numbers of seeded cells and analyzed in Western Blot **(F)**. Western blot densitometry of C3 **(G)** and CFH **(H)** from HPC-supernatants (white bars) and CiGenC-supernatants (black bars) (*n* = 6) Recombinant C3 and CFH were used as positive controls (Pos-Ctrl) in **(C,F)** in dilution 1:1,000. Data (relative expression) are shown in mean and SD in **(A,B,D,E,G,H)**, **p < 0.01, ***p < 0.001, Mann Whitney *U*-test.

Podocytes are always affected in proteinuric glomerulopathies. Nevertheless, within the glomerulus there are other cell types, which can contribute to the local complement production. Glomerular endothelial cells have direct contact with serum-based complement activation and complement products, and we have previously shown that podocyte-derived VEGF regulated expression of protective complement regulators on glomerular endothelial cells (51). To determine the cell specific production of complement proteins, we compared the expression of CFH and C3 in conditionally immortalized human glomerular endothelial cells (CiGenC) (45) and podocytes (44). Cultivated podocytes produced significantly more C3 mRNA, but less CFH mRNA compared to endothelial cells (Figures 3A,B). At the protein level for C3 and CFH, there was a slight, but non-significant, higher expression of C3 in podocytes (Figures 3C,D). The expression of CFH was significantly lower in podocytes according to protein quantification (Figures 3C,E). Complement activation may happen on any glomerular cell. Hence, the secretion of produced complement products is important. From the results in mRNA production, a comparison of secretion of complement proteins C3 and CFH showed a significant higher secretion of C3 (Figures 3F,G) and a lower secretion of CFH (Figures 3F,H) in podocytes compared to endothelial cells. Therefore, we could show that complement production and secretion profiles may differ in different intraglomerular cell types. As podocytes are the main target in PAN and in most proteinuric glomerulopathies, we further analyzed the complement expression in podocytes.

### Podocyte-Derived CFH Is Efficient in Complement Control

**Figure 4:**
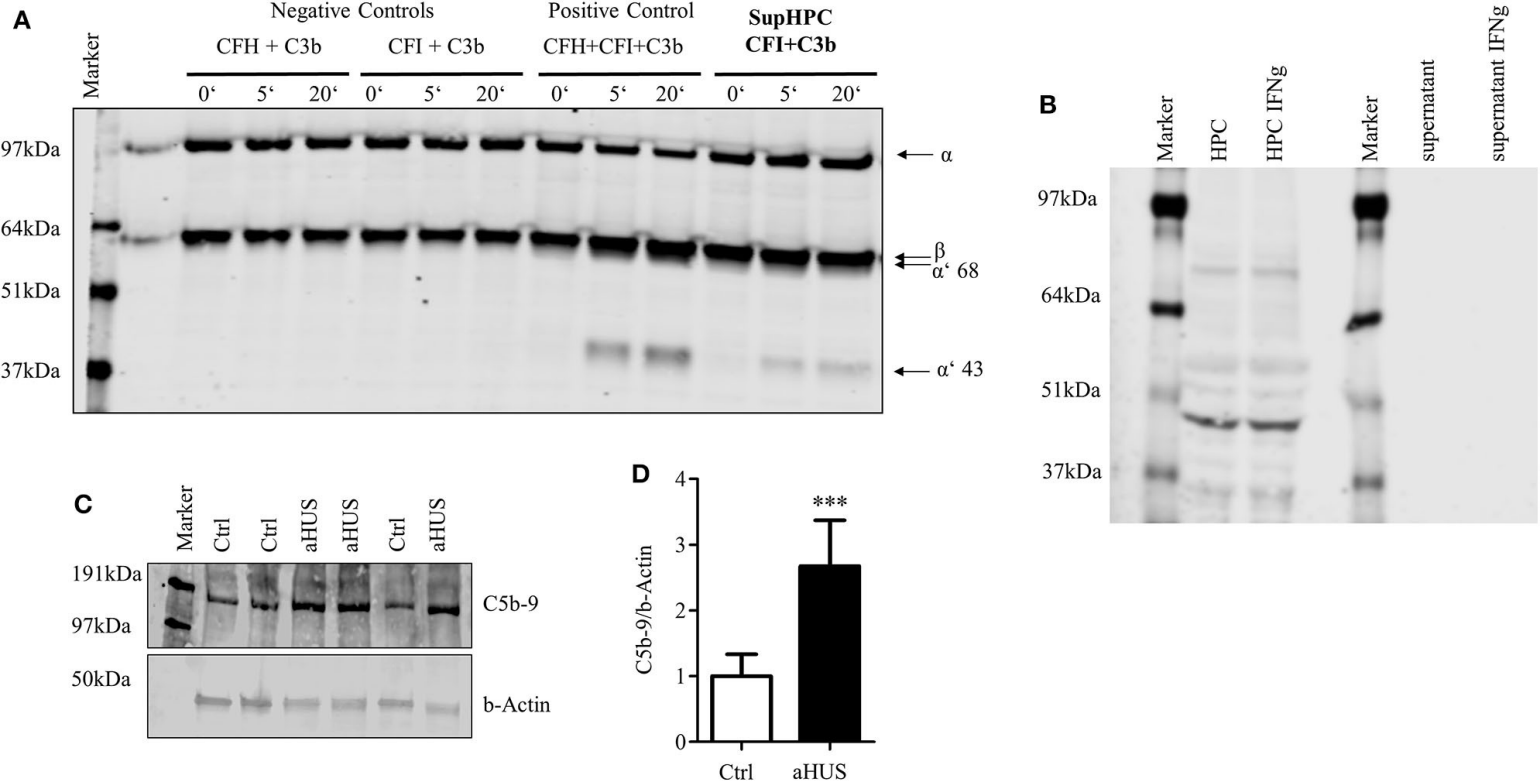
Podocyte-secreted CFH functionally active. Functionality of secreted CFH was tested in a cofactor assay and in a C5b-9 deposition assay in podocytes with lost CFH derived complement control. **(A)** Cofactor assay: CFH together with CFI can split recombinant C3b. Split products with a size of 68 and 43 kDa can be detected in Western blot against C3. Podocyte supernatant with added CFI was able to cleave added recombinant C3b. Positive control: C3b, CFI and CFH were added to serum free media (SFM) in supersaturated amounts, leading to immediate cleavage of C3b (illustrated by the appearance of the cleavage products of α′68- and α′43 kD). There is no detection of any cleavage products after the incubation of C3b and CFI, or C3b and CFI in SFM (left part). When C3b and CFI are added to podocyte supernatant, C3b split products can be detected. **(B)** Podocyte lysate (HPC) shows a clear signal for CD46 as a membrane bound complement regulator, but did not shed CD46 into the supernatant. The treatment of podocytes with interferon γ (IFNg, 10 ng/ml, 24 h) did not result in an induction of CD46 in the cell lysate or enhanced shedding in the cell culture supernatant (representative Western blot of 3 independent experiments). **(C,D)** The deposition of terminal complement complex C5b-9 was compared on normal human podocytes and podocytes from a patient suffering from atypical hemolytic uremic syndrome (aHUS). Patient's CFH was not able to bind to the cell surface. Complement activation was achieved by the induction of cell surface antigen-antibody binding followed by CFH-deficient human serum. Deposition of C5b-9 was assessed in a Western blot. **(C)** Western blot against C5b-9 on normal human podocytes (Ctrl) and aHUS patient's podocytes. **(D)** After complement activation aHUS podocytes (black bar) showed increased C5b-9 deposits compared to control cells (white bar) (relative deposition of C5b-9 related to actin, ***p < 0.001, Mann Whitney *U*-test, *n* = 4 independent experiments. Data are shown in mean and SD. **(D)** Representative Western blots of at least *n* = 3 experiments are shown in **(A–C)**.

One of the major functions of CFH is its cofactor activity in assisting CFI. With the help of CFH, CFI is able to split and inactivate C3b. The generation of C3b is a key central step in the activation of the complement system. It is required to build the C5 convertase. Therefore, the inactivation of C3b is a principal step in complement system regulation. The functional activity of secreted CFH was tested in a cofactor assay. Podocyte supernatant, which contains secreted CFH, was incubated with recombinant CFI and C3b. Together with CFI the supernatant was able to split C3b. This was evidenced by the reduction of the C3b α-chain and the detection of the cleavage products of α′68- and α′43-kD in Western blot (Figure 4A). This indicates that podocyte-derived CFH is sufficient to act as a cofactor for CFI cleaving C3b. CD46 as a membrane bound factor also is known to split C3b together with CFI. There is an ongoing discussion about shed CD46, which might in our case also act as a cofactor for CFI and might contribute to the splitting of C3b.We could never detect any CD46 in the cell culture supernatant (Figure 4B), even though there was a positive signal in the cell lysate. There was also no detectable induction of CD46 within the cell or in the supernatant after stimulation with IFNg (different to CFH). CFH seems to be crucial for the cofactor activity in the supernatant. Nevertheless, the contribution of other complement regulators cannot be excluded in this setting.

Finally, complement activation on normal human podocytes and podocytes isolated from a patient with diagnosed aHUS was compared. aHUS is a rare disorder of complement regulation, which results in kidney impairment, thrombocytopenia and anemia. A mutation in regulatory complement genes is found in many patients with this disease. We used podocytes from a patient with a known Arg1182Ser (G3546T) CFH mutation. This mutation prevents CFH from binding to the cell surface and partially to C3b (46). This is why cells from this patient cannot regulate complement activation on the surface. In a complement challenge assay, there was significantly more C5b-9 on the surface of cells containing the CFH mutation, compared to not diseased podocytes. This suggests that the secreted podocyte CFH contributed to complement regulation in the normal podocyte cell line but the mutated CFH from the aHUS cell line reduced the cells' ability to regulate complement (Figures 4C,D).

### Cultured Podocytes Can Contribute to Local Complement Activation

After the detection of C3 production in podocytes, we were interested in investigating the possibility that podocytes secrete other components of the complement cascade. In keeping with the results from Li et al. (35), we were also able to detect other components of the complement system in cultured podocytes (Supplementary Figure 1). Nevertheless, we were not able to detect mRNA for the later complement components C6, C7, C8, or C9.

Even though the podocyte could not build a complete C5b-9 complex, we wanted to study whether podocyte-secreted C3 can take part in a local extracellular complement reaction. We therefore induced complement activation on the surface of cultured rat podocytes and analyzed the sublytic complement activation. This model was used, as it is a well-characterized cell culture-based model for complement activation on podocytes. Sublytic complement activation is known to induce the phosphorylation of MAPK (58, 59). A scheme of the detailed experiment setting is shown in Supplementary Figure 2. Primary rat podocytes were incubated with anti-Fx1a antibodies, which were isolated from immunized sheep (58) to establish antibodies, which were isolated from immunized sheep (58) to establish activated with the addition of NHS. The assembly of the C5b-9 complex was shown in a Western blot. The blot showed a band in the range of C9, which probably corresponds to a neo-epitope on C9, which can be recognized by the antibody with the appearance of C5b-9. As we could see no lysis or apoptosis after this treatment, we assumed sublytic concentrations of C5b-9. To show the functionality of the assumed C5b-9 complex formation, we analyzed a well-known intracellular downstream pathway, the activation of MAPK. This pathway is very important in podocytes' differentiation and proliferation.

**Figure 5:**
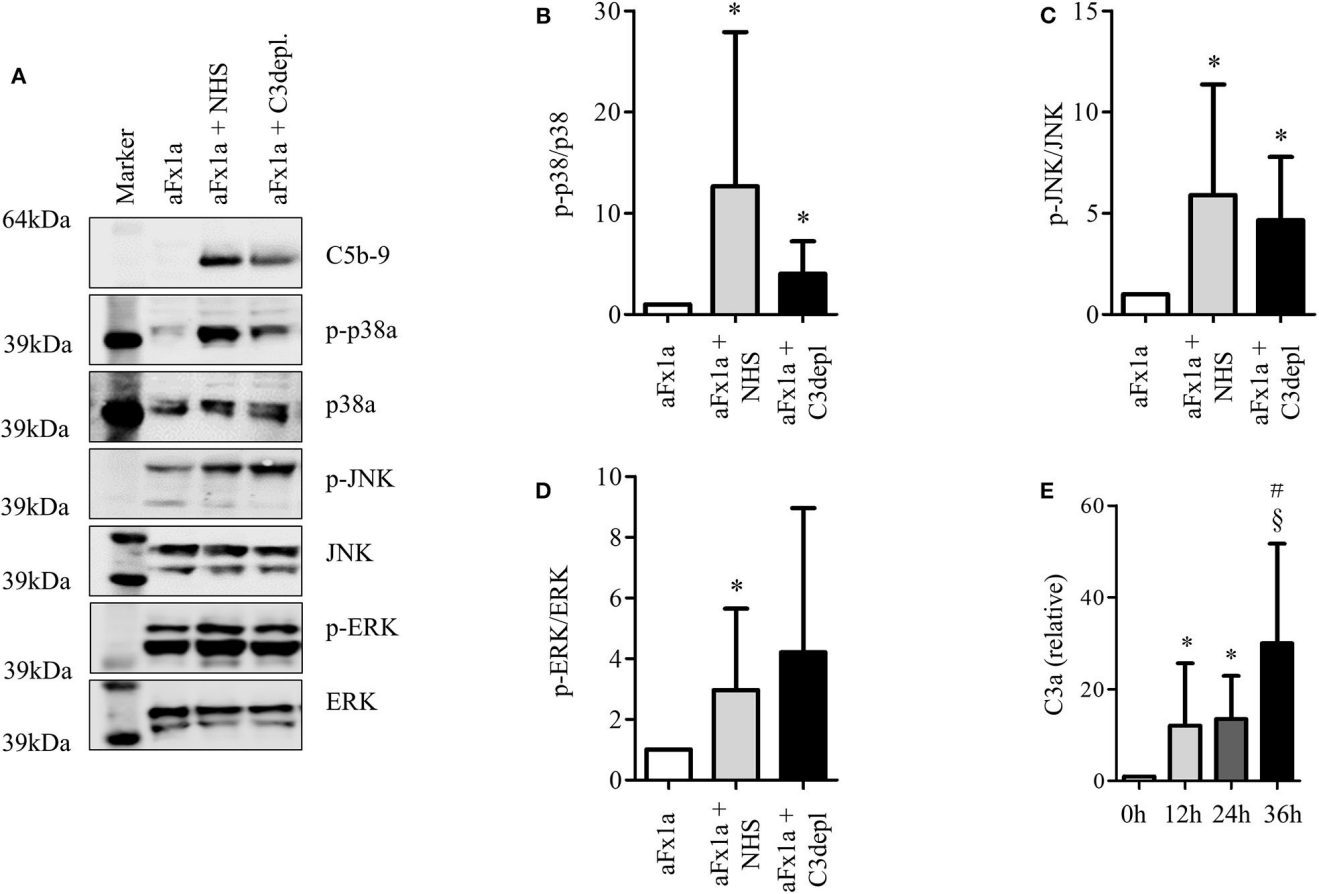
Podocyte-produced C3 contributes to complement activation. **(A–D)** Primary rat podocytes were treated with anti-Fx1a antibodies, followed by incubation with NHS as a source of complement factors. The formation of the terminal complement complex C5b-9 and the phosphorylation of mitogen activated protein kinases (MAPK), were measured in Western blots. To evaluate the influence of C3 secreted from podocytes, NHS was replaced by C3-depleted serum. Therefore, any formation of C5b-9 or phosphorylation of MAPK must involve C3 originating from podocytes. **(A)** Western blot confirming the formation of C5b-9 (first row) after treatment with anti-Fx1a antibodies and the addition of NHS or C3-depleted (depl.) serum. Both treatments induced an enhanced phosphorylation of p38a (second row), JNK (fourth row) and ERK (sixth row), while the total unphosphorylated contents of these proteins showed no changes at all (third, fifth and seventh row). Quantification of 6 independent experiments are shown in densitometry for **(B)** phosphorylated p38α, **(C)** phosphorylated JNK and **(D)** phosphorylated ERK. Data are expressed in mean and SD. Phosphorylated forms are normalized to total protein content. Mann Whitney *U*-test, *p < 0.05 compared to aFx1a. **(E)** The concentration of C3a in cell culture supernatant of unstimulated human podocytes was measured by ELISA (*p < 0.05, compared to 0 h, ^#^*p* < 0.05 compared to 12 h, ^§^*p* < 0.05 compared to 24 h, Data are shown in mean and SEM, Mann Whitney *U*-test, *n* = 5 independent experiments).

To evaluate the influence of podocyte-derived C3, C3-depleted serum was then used instead of NHS. We compared the formation of C5b-9 and downstream activation of MAPK. We confirmed the formation of C5b-9 (Figure 5A) and the phosphorylation of p38a (Figures 5A,B), JNK (Figures 5A,C), and ERK1/2 (Figures 5A,D) even in the group which was treated with C3-depleted sera. These results suggest that cultured rat podocytes are capable of secreting the missing C3 in order to complete the cascade.

Furthermore, C3a was identified in the supernatant (Figure 5E). C3a is one of the proteins formed by the cleavage of C3. C3a formation occurs through activation and cleavage of C3, which is catalyzed by C3 convertase. This indicates that, under specific circumstances, podocytes are capable of producing C3 splitting enzymes, e.g., a C3 convertase.

### C3 and CFH Are Processed in Golgi Apparatus

**Figure 6:**
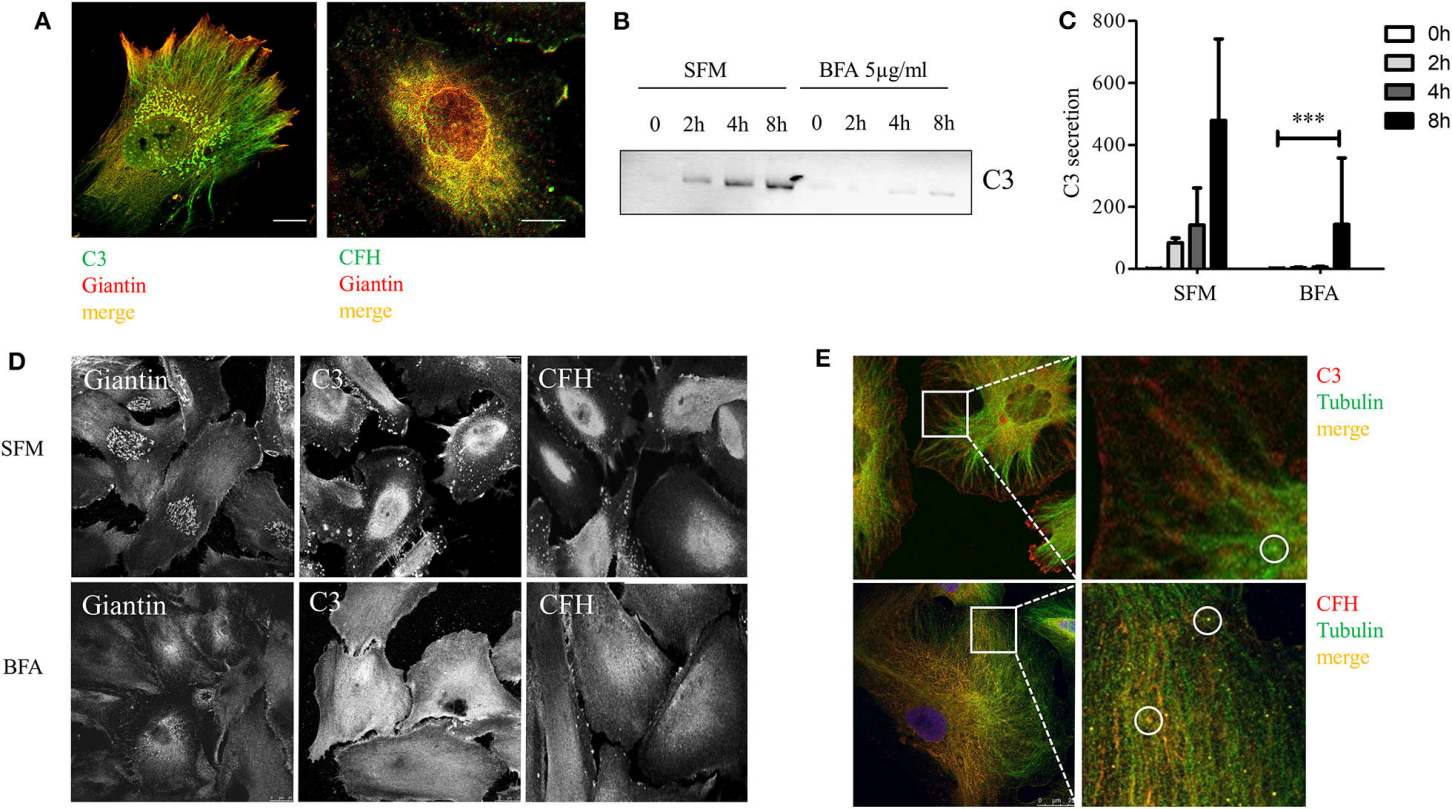
CFH and C3 were localized in the Golgi apparatus and secretion was dependent on normal Golgi function. **(A)** Podocytes were stained for C3 (left, green) and CFH (right, green) and with an antibody against giantin, a Golgi apparatus protein (red). Co-localization is shown in yellow for both proteins (40x, scale bar 25 μm). **(B,C)** Podocytes were treated with Brefeldin A (BFA), a general exocytosis inhibitor. The secretion of C3 into the cell culture supernatant was tested in Western blot, after treatment with BFA 5 μg/ml compared to SFM. **(B)** Western blot against C3 and quantification **(C)** after 0 (white bars), 2 (light gray bars), 4 (dark gray bars), and 8 h (black bars). Data are shown in mean and SD, Mann Whitney *U*-test, ***p < 0.001 compared to SFM, *n* = 3. **(D)** Podocytes were treated with BFA and stained for giantin (left panel), C3 (middle), and CFH (right) before (SFM) and after 4 h treatment: The regular Golgi structure in podocytes after incubation in SFM (above), dissolves after treatment with BFA (below). The intracellular distribution of C3 and CFH changes after BFA treatment from a mostly perinuclear (above) to a more diffuse cytoplasmic staining after the disruption of the Golgi apparatus (below). **(E)** Immunofluorescence staining of C3 (above) and CFH (below) stained in red, co-localized with staining of tubulin (green), an important component of microtubules, which regulate transportation on cells. C3 and CFH were partly localized in small round structures (circles), which might be equivalent to secretion vesicles. Representative pictures of at least *n* = 3 independent experiments are shown in **(A,D,E)**, scale bar 25 μm, 40x.

To confirm that the cultured podocyte can actively secrete complement components, we investigated the intracellular localization of C3 and CFH. Podocytes were co-stained for C3, CFH, and the Golgi apparatus marker giantin. CFH and C3 co-localized with giantin (Figure 6A, detailed split images can be seen in Supplementary Figure 3). Negative controls showed no staining (data not shown). Brefeldin A (BFA), a general inhibitor of exocytosis, leads to a retrograde transport of proteins and a disruption of the Golgi apparatus. Incubation with BFA reduced the secretion of C3 in podocytes (Figures 6B,C). The influence of BFA on CFH secretion could not be measured, because CFH was barely detectable in the supernatant before 12 h of incubation, and treatment with BFA for longer than 8 h resulted in apoptosis of these cells. Treatment with BFA for 4 h led to collapse of the Golgi stacks and C3 and CFH moved from the perinucleus to a more diffuse cytoplasmic distribution (Figure 6D). These results indicate that complement proteins are processed in the Golgi apparatus in podocytes.

To determine the specific transportation pathways, we analyzed the co-localization of complement C3 and CFH with microtubules. Microtubules play an important role in intracellular transportation pathways and can be visualized by the staining of tubulin. We found a clear co-localization of C3 and CFH with tubulin. C3 and CFH were partly found in small round structures, which might be transportation vesicles (Figure 6E). These results indicate that podocyte-produced complement proteins are processed in the Golgi apparatus, packed in secretion vesicles and moved to the surface along the microtubules.

### Glomerular C3 and CFH Is Upregulated in PAN

**Figure 7:**
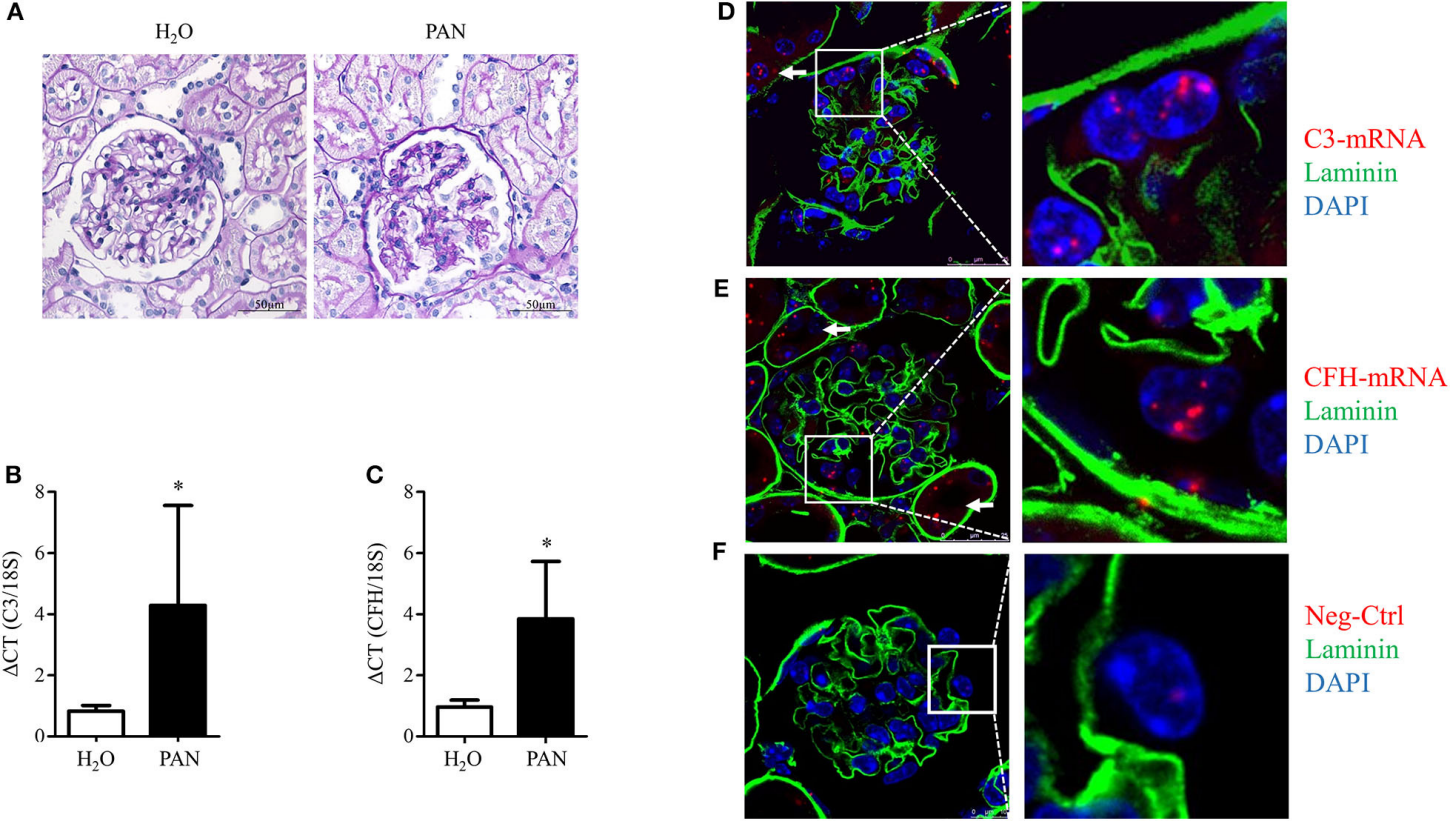
Glomerular C3- and CFH-mRNA is upregulated in puromycin aminonucleoside-induced nephropathy (PAN) in rats and C3 and CFH is expressed in glomeruli of healthy mice. Rats were treated with puromycin aminonucleoside to induce PAN. Rats were euthanized at day 28, kidneys were obtained and glomeruli were isolated. **(A)** Periodic Acid Schiff (PAS) staining in kidneys from controls (left) and PAN (right) (representative pictures, *n* = 3, scale bars 50 μm, 40x). **(B)** Expression of glomerular C3 mRNA and **(C)** glomerular CFH-mRNA in PAN (black bars) compared to controls (white bars). Ribosomal 18S-mRNA was used as an internal control and data are expressed in mean ΔCT ± SD in **(B,C)**. *p < 0.05, Mann-Whitney *U*-test; *n* = 3–5. **(D–F)** Fluorescence *in situ* hybridization of C3 **(D)**, CFH **(E)**, and a “no probe” negative control (Neg-Ctrl) in healthy mice **(F)**. C3 and CFH (red dots) co-localized with cell nuclei (blue), basement membrane was stained with laminin (green). Cells, which were outside of the basement membrane, were counted as podocytes. Podocyte expression of C3- and CFH-mRNA is shown in the enlarged frames. Arrows mark tubular expression of C3 and CFH. Figures show representative pictures of at least three independent experiments, scale bar 25 μm, 60x.

To evaluate if glomerular damage also results in the regulation of complement genes, PAN was induced in male Sprague–Dawley rats. PAN is a well-characterized and established model to mimic proteinuric diseases. Puromycin induces injury of the podocyte actin cytoskeleton (60) leading to sustained foot process effacement, podocyte loss, glomerular sclerosis, and proteinuria. As we were interested in the balance between C3, the central activating component, and CFH, the most important inhibitor of the alternative pathway, we investigated glomerular damage and mRNA expression of these two complement proteins in isolated glomeruli. After 14 days, PAN rats developed severe proteinuria (data not shown). Staining with Periodic Schiff Acid showed massive sclerosis and glomerular damage (Figure 7A). In these studies, we confirmed the results of Li et al. (35), concerning the glomerular upregulation of C3 mRNA in PAN (Figure 7B). In addition, we detected an increased induction of CFH in comparison to H_2_O-treated controls (Figure 7C). As podocytes are the main target in PAN, we were extremely interested in the ability of podocytes to produce these critical opposing players in the complement system.

### Podocytes Expressed CFH and C3 *in vivo*

Unfortunately, we were not able to show any positive staining for C3 or CFH protein in podocytes in murine or human tissue (data not shown). Therefore, to prove the origin of CFH and C3 production in glomeruli *in vivo*, fluorescence *in situ* hybridization (FISH) studies were performed in glomeruli of healthy C57Bl/6 mice. C3- and CFH-mRNA was identified in glomeruli of healthy C57Bl/6 mice (Figures 7D,E). Negative control showed no staining of the mRNA signal (Figure 7F). The mRNA of C3 and CFH partially co-localized with podocyte nuclei. These findings are clear evidence that local glomerular complement production occurs within the glomeruli and in podocytes. Furthermore, there was C3 and CFH in tubular cells, indicating that podocytes are not the only source in the kidney.

## Discussion

Podocytes are complex differentiated cells that are essential for the functionality of the glomerular filtration barrier and renal function. Damaged podocytes have very limited potential for recovery and regeneration (61). Therefore, the integrity of the podocyte and the resistance against damage due to local complement activation or against invading pathogens is important. The complement system is a crucial part of the innate immune system. It helps with the elimination of invading microorganisms and the removal of apoptotic cells. Nevertheless, it has also been shown to contribute to a growing list of inflammatory conditions (62). The kidney is particularly sensitive to complement-mediated damage. In several systemic complement disorders, for example in diseases like aHUS or systemic lupus erythematosus (SLE), the kidney is a particular target. The reason for the kidney's unique susceptibility to complement-induced damage is not yet fully elucidated, but it could be influenced by its structural organization and functional duties (63). Renal perfusion contributes to the constant contact of the glomerulus with several circulating toxins or immune cells.

With the podocyte at the center of all forms of proteinuria, these cells were analyzed to determine their potential to produce complement proteins. Our studies showed that podocytes not only express and store complement proteins, they can also secrete functionally active C3 and CFH, which is enhanced by IFNg, an immune-stimulating protein. Therefore, it is probable that podocytes influence local complement activation in situations of immune-related challenge, for example in infection and inflammation. The need for resistance of kidney cells against invading microorganisms might be a good explanation for kidney cells' potential to express complement activating components. The kidney seems to have an activated complement profile under unstimulated conditions. These complement proteins might serve to remove immune complexes and provide protection against microorganisms of the urinary tract (64). Podocytes, sitting on the outside of the glomerular filtration barrier, are especially prone to contact with invasive pathogenic substances. Therefore, the expression of C3, as the key player in complement activation and particularly the secretion of C3a as a potent anaphylatoxin, could be crucially useful. On the other hand, the podocyte-produced C3 could also contribute to activation of the system due to a systemic complement disorder or secondary complement activation in inflammatory circumstances. This might explain why podocytes are damaged in complement mediated diseases like C3 glomerulonephritis or aHUS.

It has been described previously how several complement components were locally produced by parenchymal cells of the glomeruli and in tubules. Li et al. (35) have already shown a glomerular C3 upregulation in an experimental proteinuric model. We confirmed their findings that podocytes are one source of C3 mRNA using FISH and indirectly via glomerular upregulation in PAN treated rats. We also showed that podocytes in culture are able to actively secrete C3, which contributed to external experimental complement activation. The detection of C3a indicated that podocyte-secreted C3 is functionally active and can induce complement activation. This might occur during local inflammatory conditions. With the generation of C3 and C3a, podocytes could activate complement upstream of C5b-9. Additionally, the secreted complement proteins could be washed into the urinary space. Several complement proteins have already been detected in urine (14, 65–72). The occurring protein leak in situations of proteinuria was previously thought to be responsible for this detection. Others have suspected production of C3 by tubular cells (73). Our work also detection. Others have suspected production of C3 by tubular cells (73). Our work As has already been shown, kidney cells, and podocytes in particular, are affected directly by complement activation (74, 75). Therefore, defending mechanisms are essential to prevent permanent damage. Podocytes, like several other cells, produce membrane-bound regulators to protect themselves from complement attack (76). Membrane-bound regulators, such as CD46, CD55, and CD59, protect the respective regulator-expressing cell from the assembling of complement activation products on the cell surface. Additionally, there are circulating complement regulators like CFH, which predominantly regulate complement activation in the alternative pathway. CFH molecules are mainly produced in the liver. However, we also detected the expression of CFH *in vitro* and *in vivo* in podocytes. The podocyte-derived CFH acted as a cofactor for CFI to cleave C3b in a cofactor assay, showing that it was functionally active. A very important other mechanism of CFH in complement control is the decay of the convertase, which was not tested in detail in this study. Nevertheless, in a setting with functionally defective CFH, podocytes were not able to prevent the adhesion of C5b-9 during the complement attack.

It could be argued that aHUS is exclusively an endothelial cell disorder, but proteinuria can be a presenting clinical feature of this disease, confirming that podocytes are affected early in the disease (77). The detection of impaired podocyte CFH in one aHUS patient (78) supports the importance of podocyte-derived CFH in protecting the glomerulum from complement mediated injury. Further, other researchers have recently suggested that podocyte dysfunction can occur in acquired and genetic forms of complement-mediated aHUS, suggesting that podocytes do indeed play a role in this disease (79). Loss of local complement regulation may be important for those patients who require a second hit before manifestation of the disease. The local production of CFH also seems to be essential in SLE, where it is responsible for processing the immune complexes. The deficiency of CFH results in an accelerated development of lupus nephritis in an animal model for SLE (80). Furthermore, an important role of podocyte derived CFH development of lupus nephritis in an animal model for SLE (80). Furthermore, an Another indication that the podocyte is a local source for CFH is the detection of CFH in urine from healthy individuals (81). CFH, a 150 kDa serum protein, would not normally undergo ultrafiltration. In this context, CFH may be produced on demand locally and play a regulatory role in urine even under normal circumstances. There is evidence for the local importance of CFH in other diseases as well. In membranous nephropathy, the ratio of urinary C5b-9 to CFH was associated with active glomerular disease highlighting the protective role of this protein in disease (81). Elevated urinary CFH levels have also been detected in other glomerulopathies (70, 82). In our opinion podocytes, in addition to other glomerular cells, are one source of CFH. Another source of this urinary CFH could be the tubular cells, which showed a positive CFH and C3 mRNA signal in FISH. Nevertheless, in glomerular diseases tubular cells are usually damaged later, while proteinuria indicates that glomerular cells are affected early on. This local glomerular interplay could be important concerning CFH production, since it has a complement regulatory role on basement membranes that do not express other complement regulators (83).

Endothelial cells in culture can produce and secrete complement factors such as C3 and CFH and can contribute to local glomerular complement activation. However, there was a variation in the level of expression in both factors between podocytes and glomerular endothelial cells, suggesting distinct levels of regulation within different sections of the glomerulus. Even though this different expression level was described only *in vitro*, it indicates that glomerular cells might have different individual tasks. We could hypothesize that they have to work together to organize appropriate local complement activation. These glomerular cell types have to ensure that there is a distinct balance between activation and regulation of the cascade to prevent uncontrolled kidney damage. Our previous work has shown that there is a synergistic role between podocytes and endothelial cells in glomerular complement regulation (51).

The different expression levels of complement factors lead to another additional consideration: it has already been shown that there is an important crosstalk between podocytes and endothelial cells, which occurs particularly with regards to VEGF (4). On one hand, the production of different factors in different cells could contribute to complement activation on different levels. On the other hand, the secretion of complement factors might have additional novel roles for other surrounding cells. There is a growing body of evidence that C3 and C3a have roles extending beyond those of complement system activation. Rather than being just a pro-inflammatory effector system, complement is emerging as a central player in cell differentiation, proliferation, repair and induction in T-cells (84). The complement system is not only thought to repair and induction in T-cells (84). The complement system is not only thought intracellular complement—the so called *complosome*—seems to have an impact on normal cell physiology (85). Nevertheless, the meaning of local on normal cell physiology (85). Nevertheless, the meaning of local endothelial cells has not yet been uncovered. Even though, the podocyte might not be the only or main source of kidney derived complement proteins, it is not known, whether even low amounts of locally produced activating or regulating factors might have a relatively higher impact on local inflammation. The hypothesis, why we were not able to show any positive protein signal for C3 or CFH, might be due to the low expression of both proteins in podocytes compared to other cell-types or the concentration measured systemically in the blood. Another explanation might be that both proteins are not stored in huge amounts within the cells and might be secreted rapidly to regulate the local complement activation.

However, one of the most important conclusions resulting from our findings is that the podocyte seems to have more tasks than just mechanistic stabilization of the glomerular filter. There is a growing body of evidence that podocytes may act as immune modulating cells (38). Even though they are affected in this disease themselves, podocytes seem to play an important role in the regulation of complement factors that may prevent nephritis in SLE; they also influence innate and adaptive immunity. It has been shown that isolated glomeruli express TLRs, for example TLR4 (40), which is associated with the development of lupus nephritis. The activation of TLR4 (e.g., with lipopolysaccharide) induces the expression of various cytokines (e.g., CCL2, CCL7, CXCL1, CXCL5, CCL3, CCL5, and more) (37, 38). Therefore, the podocyte seems to be able to create an inflammatory milieu in certain circumstances. Additionally, podocytes express the receptor for advanced glycation end products (RAGE), which is important in several immune reactions and which is upregulated in ADR-induced nephropathy (86). They are also capable of expressing the NLRP3 inflammasome, which is activated in the development of proteinuria in lupus nephritis (87).

With our findings we demonstrated that podocytes have a role beyond a purely stabilization function in the glomerulus. The podocyte might act as an immune modulating, and especially local complement modulating, cell. In states of proteinuria, primary or secondary complement activation can occur within the glomeruli and podocytes seem to be able to contribute to this complement activation by production of activating or inhibitory complement components. Even though there is increasing knowledge about the involvement of complement in kidney diseases, the new role of the podocyte as the local source of complement activators and regulators requires additional study in all causes of glomerular complement activation. Furthermore, our results underline a possible role of the podocyte as an immune modulating cell.

## Data Availability Statement

The datasets generated for this study are available on request to the corresponding author.

## Ethics Statement

The animal study was reviewed and approved by Behörde für Gesundheit und Verbraucherschutz, Fachbereich Veterinärwesen.

## Author Contributions

AM, LK, CL, JO, and MS contributed conception and design of the study. AM, LK, JA, and HH performed the experiments. RH, GW, CM-S, RC, and LF designed parts of the experiments and wrote sections of the manuscript. All authors contributed to the manuscript revision, creation of the final manuscript, read, and approved the submitted version.

## Conflict of Interest

The authors declare that the research was conducted in the absence of any commercial or financial relationships that could be construed as a potential conflict of interest.

## Supplementary Material

Supplementary Figure 1 Unstimulated human podocytes (HPC) express mRNA for various complement factors in conventional PCR (-RT, control of cDNA without addition of RT; bp, base pairs).

Supplementary Figure 2 Schematic presentation of the assumed underlying mechanisms in the complement challenge assay. **(A)** The binding of anti-podocyte antibodies leads to the activation of the complement cascade via the classical pathway (after the addition of normal human serum as a complete source of complement factors). The complement activation and building of C5b-9 induces sub-lytic damage in this *in vitro* model, which can be shown with the detection of phosphorylation of mitogen activated psrotein kinases (MAPK). **(B)** The use of C3-depleted serum did not prevent the phosphorylation of MAPK, which proves that podocytes derived C3 replaces external C3.

Supplementary Figure 3 Detailed spilt images: Podocytes were stained for C3 and CFH (green) and with an antibody against giantin, a Golgi apparatus protein (red). Co-localization is shown in yellow for both proteins (40x, scale bar 25 μm).

Supplementary Table 1 Primer sequences conventional PCR, primers were obtained from Sigma Aldrich.

## References

[1] Woolhandler S Pels RJ Bor DH Himmelstein DU Lawrence RS. Dipstick urinalysis screening of asymptomatic adults for urinary tract disorders. I Hematuria and proteinuria. JAMA. (1989) 262:1214–9. 2668582

[2] Wenderfer SE Gaut JP. Glomerular diseases in children. Adv Chronic Kidney Dis. (2017) 24:364–71. 10.1053/j.ackd.2017.09.005 29229167

[3] Viteri B Reid-Adam J. Hematuria and proteinuria in children. Pediatr Rev. (2018) 39:573–87. 10.1542/pir.2017-0300 30504250 PMC6494107

[4] Eremina V Jefferson JA Kowalewska J Hochster H Haas M Weisstuch J. VEGF inhibition and renal thrombotic microangiopathy. N Engl J Med. (2008) 358:1129–36. 10.1056/NEJMoa0707330 18337603 PMC3030578

[5] Büscher AK Weber S. Educational paper: the podocytopathies. Eur J Pediatr. (2012) 171:1151–60. 10.1007/s00431-011-1668-2 22237399

[6] Ma H Sandor DG Beck LH. The role of complement in membranous nephropathy. Semin Nephrol. (2013) 33:531–42. 10.1016/j.semnephrol.2013.08.004 24161038 PMC4274996

[7] Keir L Coward RJ. Advances in our understanding of the pathogenesis of glomerular thrombotic microangiopathy. Pediatr Nephrol. (2011) 26:523–33. 10.1007/s00467-010-1637-4 20949284 PMC3043262

[8] Liszewski MK Kolev M Le Friec G Leung M Bertram PG Fara AF. Intracellular complement activation sustains T cell homeostasis and mediates effector differentiation. Immunity. (2013) 39:1143–57. 10.1016/j.immuni.2013.10.018 24315997 PMC3865363

[9] Pangburn MK. Host recognition and target differentiation by factor H, a regulator of the alternative pathway of complement. Immunopharmacology. (2000) 49:149–57. 10.1016/s0162-3109(00)80300-8 10904114

[10] Pickering MC Cook HT. Translational mini-review series on complement factor H: renal diseases associated with complement factor H: novel insights from humans and animals. Clin Exp Immunol. (2008) 151:210–30. 10.1111/j.1365-2249.2007.03574.x 18190458 PMC2276951

[11] Goodship TH. Atypical HUS and complement dysregulation. J Am Soc Nephrol. (2006) 17:1775–6. 10.1681/ASN.2006050521 16790505

[12] Pickering MC Cook HT Warren J Bygrave AE Moss J Walport MJ. Uncontrolled C3 activation causes membranoproliferative glomerulonephritis in mice deficient in complement factor H. Nat Genet. (2002) 31:424–8. 10.1038/ng912 12091909

[13] Tortajada A Yébenes H Abarrategui-Garrido C Anter J García-Fernández JM Martínez-Barricarte R. C3 glomerulopathy-associated CFHR1 mutation alters FHR oligomerization and complement regulation. J Clin Invest. (2013) 123:2434–46. 10.1172/JCI68280 23728178 PMC3668852

[14] Maillard N Wyatt RJ Julian BA Kiryluk K Gharavi A Fremeaux-Bacchi V. Current Understanding of the role of complement in IgA nephropathy. J Am Soc Nephrol. (2015) 26:1503–12. 10.1681/ASN.2014101000 25694468 PMC4483595

[15] Keir LS Langman CB. Complement and the kidney in the setting of Shiga-toxin hemolytic uremic syndrome, organ transplantation, and C3 glomerulonephritis. Transfus Apher Sci. (2016) 54:203–11. 10.1016/j.transci.2016.04.010 27156109

[16] Andoh A Fujiyama Y Bamba T Hosoda S. Differential cytokine regulation of complement C3, C4, and factor B synthesis in human intestinal epithelial cell line, Caco-2. J Immunol. (1993) 151:4239–47. 8409399

[17] Andoh A Fujiyama Y Sumiyoshi K Bamba T. Local secretion of complement C3 in the exocrine pancreas: ductal epithelial cells as a possible biosynthetic site. Gastroenterology. (1996) 110:1919–25. 8964419 10.1053/gast.1996.v110.pm8964419

[18] Andoh A Fujiyama Y Sakumoto H Uchihara H Kimura T Koyama S. Detection of complement C3 and factor B gene expression in normal colorectal mucosa, adenomas and carcinomas. Clin Exp Immunol. (1998) 111:477–83. 9528886 10.1046/j.1365-2249.1998.00496.x PMC1904873

[19] Andoh A Shimada M Takaya H Hata K Fujiyama Y Bamba T. Transforming growth factor-beta1 acts as a potent inhibitor of complement C3 biosynthesis in human pancreatic cancer cell lines. Pancreas. (2000) 20:138–45. 10.1097/00006676-200003000-00005 10707928

[20] Li K Fazekasova H Wang N Sagoo P Peng Q Khamri W. Expression of complement components, receptors and regulators by human dendritic cells. Mol Immunol. (2011) 48:1121–7. 10.1016/j.molimm.2011.02.003 21397947 PMC3084445

[21] Naughton MA Botto M Carter MJ Alexander GJ Goldman JM Walport MJ. Extrahepatic secreted complement C3 contributes to circulating C3 levels in humans. J Immunol. (1996) 156:3051–6. 8609428

[22] Feucht HE Zwirner J Bevec D Lang M Felber E Riethmuller G. Biosynthesis of complement C4 messenger RNA in normal human kidney. Nephron. (1989) 53:338–42. 2601801 10.1159/000185778

[23] Sacks SH Zhou W Andrews PA Hartley B. Endogenous complement C3 synthesis in immune complex nephritis. Lancet. (1993) 342:1273–4. 7901586 10.1016/0140-6736(93)92362-w

[24] Miyazaki M Abe K Koji T Furusu A Ozono Y Harada T. Intraglomerular C3 synthesis in human kidney detected by *in situ* hybridization. J Am Soc Nephrol. (1996) 7:2428–33. 8959636 10.1681/ASN.V7112428

[25] Serinsöz E Bock O Gwinner W Schwarz A Haller H Kreipe H. Local complement C3 expression is upregulated in humoral and cellular rejection of renal allografts. Am J Transplant. (2005) 5:1490–4. 10.1111/j.1600-6143.2005.00873.x 15888059

[26] Farrar CA Zhou W Lin T Sacks SH. Local extravascular pool of C3 is a determinant of postischemic acute renal failure. FASEB J. (2006) 20:217–26. 10.1096/fj.05-4747com 16449793

[27] Damman J Seelen MA Moers C Daha MR Rahmel A Leuvenink HG. Systemic complement activation in deceased donors is associated with acute rejection after renal transplantation in the recipient. Transplantation. (2011) 92:163–9. 10.1097/TP.0b013e318222c9a0 21677599

[28] Sacks S Zhou W Campbell RD Martin J. C3 and C4 gene expression and interferon-gamma-mediated regulation in human glomerular mesangial cells. Clin Exp Immunol. (1993) 93:411–7. 8370168 10.1111/j.1365-2249.1993.tb08193.x PMC1554924

[29] Sacks SH Zhou W Pani A Campbell RD Martin J. Complement C3 gene expression and regulation in human glomerular epithelial cells. Immunology. (1993) 79:348–54. 8406564 PMC1421987

[30] Zhou W Campbell RD Martin J Sacks SH. Interferon-gamma regulation of C4 gene expression in cultured human glomerular epithelial cells. Eur J Immunol. (1993) 23:2477–81. 10.1002/eji.1830231015 8405048

[31] Sheerin NS Zhou W Adler S Sacks SH. TNF-alpha regulation of C3 gene expression and protein biosynthesis in rat glomerular endothelial cells. Kidney Int. (1997) 51:703–10. 9067902 10.1038/ki.1997.101

[32] Zhou W Marsh JE Sacks SH. Intrarenal synthesis of complement. Kidney Int. (2001) 59:1227–35. 10.1046/j.1523-1755.2001.0590041227.x 11260382

[33] Timmerman JJ van der Woude FJ van Gijlswijk-Janssen DJ Verweij CL van Es LA Daha MR. Differential expression of complement components in human fetal and adult kidneys. Kidney Int. (1996) 49:730–40. 8648914 10.1038/ki.1996.102

[34] Sheerin NS Risley P Abe K Tang Z Wong W Lin T. Synthesis of complement protein C3 in the kidney is an important mediator of local tissue injury. FASEB J. (2008) 22:1065–72. 10.1096/fj.07-8719com 18039928

[35] Li X Ding F Zhang X Li B Ding J. The expression profile of complement components in podocytes. Int J Mol Sci. (2016) 17:471. 10.3390/ijms17040471 27043537 PMC4848927

[36] Zoshima T Hara S Yamagishi M Pastan I Matsusaka T Kawano M. Possible role of complement factor H in podocytes in clearing glomerular subendothelial immune complex deposits. Sci Rep. (2019) 9:7857. 10.1038/s41598-019-44380-3 31133737 PMC6536504

[37] Xia H Bao W Shi S. Innate immune activity in glomerular podocytes. Front Immunol. (2017) 8:122. 10.3389/fimmu.2017.00122 28228761 PMC5296344

[38] Bhargava R Tsokos GC. The immune podocyte. Curr Opin Rheumatol. (2019) 31:167–74. 10.1097/BOR.0000000000000578 30562182

[39] Reiser J von Gersdorff G Loos M Oh J Asanuma K Giardino L. Induction of B7-1 in podocytes is associated with nephrotic syndrome. J Clin Invest. (2004) 113:1390–7. 10.1172/JCI20402 15146236 PMC406528

[40] Banas MC Banas B Hudkins KL Wietecha TA Iyoda M Bock E. TLR4 links podocytes with the innate immune system to mediate glomerular injury. J Am Soc Nephrol. (2008) 19:704–13. 10.1681/ASN.2007040395 18256364 PMC2390962

[41] Machida H Ito S Hirose T Takeshita F Oshiro H Nakamura T. Expression of Toll-like receptor 9 in renal podocytes in childhood-onset active and inactive lupus nephritis. Nephrol Dial Transplant. (2010) 25:2530–7. 10.1093/ndt/gfq058 20181802

[42] Sheng X Zuo X Liu X Zhou Y Sun X. Crosstalk between TLR4 and Notch1 signaling in the IgA nephropathy during inflammatory response. Int Urol Nephrol. (2018) 50:779–85. 10.1007/s11255-017-1760-2 29230705

[43] Meyer-Schwesinger C Lange C Brocker V Agustian P Lehmann U Raabe A. Bone marrow-derived progenitor cells do not contribute to podocyte turnover in the puromycin aminoglycoside and renal ablation models in rats. Am J Pathol. (2011) 178:494–9. 10.1016/j.ajpath.2010.10.024 21281782 PMC3069901

[44] Saleem MA O'Hare MJ Reiser J Coward RJ Inward CD Farren T. A conditionally immortalized human podocyte cell line demonstrating nephrin and podocin expression. J Am Soc Nephrol. (2002) 13:630–8. 11856766 10.1681/ASN.V133630

[45] Satchell SC Tasman CH Singh A Ni L Geelen J von Ruhland CJ. Conditionally immortalized human glomerular endothelial cells expressing fenestrations in response to VEGF. Kidney Int. (2006) 69:1633–40. 10.1038/sj.ki.5000277 16557232

[46] Kajander T Lehtinen MJ Hyvarinen S Bhattacharjee A Leung E Isenman DE. Dual interaction of factor H with C3d and glycosaminoglycans in host-nonhost discrimination by complement. Proc Natl Acad Sci USA. (2011) 108:2897–902. 10.1073/pnas.1017087108 21285368 PMC3041134

[47] Dettmar AK Binder E Greiner FR Liebau MC Kurschat CE Jungraithmayr TC. Protection of human podocytes from shiga toxin 2-induced phosphorylation of mitogen-activated protein kinases and apoptosis by human serum amyloid P component. Infect Immun. (2014) 82:1872–9. 10.1128/IAI.01591-14 24566618 PMC3993451

[48] Keir LS Firth R May C Ni L Welsh GI Saleem MA. Generating conditionally immortalised podocyte cell lines from wild-type mice. Nephron. (2015) 129:128–36. 10.1159/000369816 25720381

[49] Meri T Blom AM Hartmann A Lenk D Meri S Zipfel PF. The hyphal and yeast forms of *Candida albicans* bind the complement regulator C4b-binding protein. Infect Immun. (2004) 72:6633–41. 10.1128/IAI.72.11.6633-6641.2004 15501796 PMC523010

[50] Heinen S Józsi M Hartmann A Noris M Remuzzi G Skerka C. Hemolytic uremic syndrome: a factor H mutation (E1172Stop) causes defective complement control at the surface of endothelial cells. J Am Soc Nephrol. (2007) 18:506–14. 10.1681/ASN.2006091069 17229916

[51] Keir LS Firth R Aponik L Feitelberg D Sakimoto S Aguilar E. VEGF regulates local inhibitory complement proteins in the eye and kidney. J Clin Invest. (2017) 127:199–214. 10.1172/JCI86418 27918307 PMC5199702

[52] Meyer-Schwesinger C Meyer TN Sievert H Hoxha E Sachs M Klupp EM. Ubiquitin C-terminal hydrolase-l1 activity induces polyubiquitin accumulation in podocytes and increases proteinuria in rat membranous nephropathy. Am J Pathol. (2011) 178:2044–57. 10.1016/j.ajpath.2011.01.017 21514420 PMC3081162

[53] Amdahl H Jarva H Haanperä M Mertsola J He Q Jokiranta TS. Interactions between Bordetella pertussis and the complement inhibitor factor H. Mol Immunol. (2011) 48:697–705. 10.1016/j.molimm.2010.11.015 21167605

[54] Nam EJ Park PW. Shedding of cell membrane-bound proteoglycans. Methods Mol Biol. (2012) 836:291–305. 10.1007/978-1-61779-498-8_19 22252642 PMC3569011

[55] Brooimans RA van der Ark AA Buurman WA van Es LA Daha MR. Differential regulation of complement factor H and C3 production in human umbilical vein endothelial cells by IFN-gamma and IL-1. J Immunol. (1990) 144:3835–40. 2139673

[56] Gerritsma JS Gerritsen AF De Ley M van Es LA Daha MR. Interferon-gamma induces biosynthesis of complement components C2, C4 and factor H by human proximal tubular epithelial cells. Cytokine. (1997) 9:276–83. 10.1006/cyto.1996.0164 9112336

[57] Luo W Vik DP. Regulation of complement factor H in a human liver cell line by interferon-gamma. Scand J Immunol. (1999) 49:487–94. 10320641 10.1046/j.1365-3083.1999.00528.x

[58] Peng H Takano T Papillon J Bijian K Khadir A Cybulsky AV. Complement activates the c-Jun N-terminal kinase/stress-activated protein kinase in glomerular epithelial cells. J Immunol. (2002) 169:2594–601. 10.4049/jimmunol.169.5.2594 12193730

[59] Aoudjit L Stanciu M Li H Lemay S Takano T. p38 mitogen-activated protein kinase protects glomerular epithelial cells from complement-mediated cell injury. Am J Physiol Renal Physiol. (2003) 285:F765–74. 10.1152/ajprenal.00100.2003 12837681

[60] Coers W Huitema S van der Horst ML Weening JJ Puromycin aminonucleoside and adriamycin disturb cytoskeletal and extracellular matrix protein organization, but not protein synthesis of cultured glomerular epithelial cells. Exp Nephrol. (1994) 2:40–50. 8081996

[61] Kriz W. Progressive renal failure–inability of podocytes to replicate and the consequences for development of glomerulosclerosis. Nephrol Dial Transplant. (1996) 11:1738–42. 8918614

[62] Reis ES Mastellos DC Yancopoulou D Risitano AM Ricklin D Lambris JD. Applying complement therapeutics to rare diseases. Clin Immunol. (2015) 161:225–40. 10.1016/j.clim.2015.08.009 26341313 PMC4658209

[63] Zoja C Abbate M Remuzzi G. Progression of renal injury toward interstitial inflammation and glomerular sclerosis is dependent on abnormal protein filtration. Nephrol Dial Transplant. (2015) 30:706–12. 10.1093/ndt/gfu261 25087196

[64] McCullough JW Renner B Thurman JM. The role of the complement system in acute kidney injury. Semin Nephrol. (2013) 33:543–56. 10.1016/j.semnephrol.2013.08.005 24161039 PMC3816009

[65] Cumming AD Thomson D Davidson AM Robson JS. Significance of urinary C3 excretion in glomerulonephritis. J Clin Pathol. (1976) 29:601–7. 789406 10.1136/jcp.29.7.601 PMC476125

[66] Klajman A Avital A Myers BD. Renal handling of the third (C3) and fourth (C4) components of the complement system in the nephrotic syndrome. Nephron. (1976) 16:333–43. 1264308 10.1159/000180620

[67] Morita Y Ikeguchi H Nakamura J Hotta N Yuzawa Y Matsuo S. Complement activation products in the urine from proteinuric patients. J Am Soc Nephrol. (2000) 11:700–7. 10752529 10.1681/ASN.V114700

[68] Negi VS Aggarwal A Dayal R Naik S Misra R. Complement degradation product C3d in urine: marker of lupus nephritis. J Rheumatol. (2000) 27:380–3. 10685801

[69] Li K Sacks SH Sheerin NS. The classical complement pathway plays a critical role in the opsonisation of uropathogenic *Escherichia coli*. Mol Immunol. (2008) 45:954–62. 10.1016/j.molimm.2007.07.037 17870166

[70] Zhang JJ Jiang L Liu G Wang SX Zou WZ Zhang H. Levels of urinary complement factor H in patients with IgA nephropathy are closely associated with disease activity. Scand J Immunol. (2009) 69:457–64. 10.1111/j.1365-3083.2009.02234.x 19508377

[71] Onda K Ohsawa I Ohi H Tamano M Mano S Wakabayashi M. Excretion of complement proteins and its activation marker C5b-9 in IgA nephropathy in relation to renal function. BMC Nephrol. (2011) 12:64. 10.1186/1471-2369-12-64 22111871 PMC3283454

[72] Kalantari S Rutishauser D Samavat S Nafar M Mahmudieh L Rezaei-Tavirani M. Urinary prognostic biomarkers and classification of IgA nephropathy by high resolution mass spectrometry coupled with liquid chromatography. PLoS ONE. (2013) 8:e80830. 10.1371/journal.pone.0080830 24339887 PMC3855054

[73] Montinaro V Lopez A Monno R Cappiello V Manno C Gesualdo L. Renal C3 synthesis in idiopathic membranous nephropathy: correlation to urinary C5b-9 excretion. Kidney Int. (2000) 57:137–46. 10.1046/j.1523-1755.2000.00812.x 10620195

[74] Takano T Cybulsky AV Cupples WA Ajikobi DO Papillon J Aoudjit L. Inhibition of cyclooxygenases reduces complement-induced glomerular epithelial cell injury and proteinuria in passive Heymann nephritis. J Pharmacol Exp Ther. (2003) 305:240–9. 10.1124/jpet.102.043604 12649375

[75] Zoja C Buelli S Morigi M. Shiga toxin triggers endothelial and podocyte injury: the role of complement activation. Pediatr Nephrol. (2017) 34:379–88. 10.1007/s00467-017-3850-x 29214442

[76] Noris M Remuzzi G. Overview of complement activation and regulation. Semin Nephrol. (2013) 33:479–92. 10.1016/j.semnephrol.2013.08.001 24161035 PMC3820029

[77] Sellier-Leclerc AL Fremeaux-Bacchi V Dragon-Durey MA Macher MA Niaudet P Guest G. Differential impact of complement mutations on clinical characteristics in atypical hemolytic uremic syndrome. J Am Soc Nephrol. (2007) 18:2392–400. 10.1681/ASN.2006080811 17599974

[78] Vaziri-Sani F Holmberg L Sjöholm AG Kristoffersson AC Manea M Frémeaux-Bacchi V. Phenotypic expression of factor H mutations in patients with atypical hemolytic uremic syndrome. Kidney Int. (2006) 69:981–8. 10.1038/sj.ki.5000155 16528247

[79] Noris M Mele C Remuzzi G. Podocyte dysfunction in atypical haemolytic uraemic syndrome. Nat Rev Nephrol. (2015) 11:245–52. 10.1038/nrneph.2014.250 25599621

[80] Bao L Haas M Quigg RJ. Complement factor H deficiency accelerates development of lupus nephritis. J Am Soc Nephrol. (2011) 22:285–95. 10.1681/ASN.2010060647 21148254 PMC3029901

[81] Endo M Fuke Y Ohi H Satomura A Fukuda N Fujita T. [Evaluation of urinary factor H excretion in patients with idiopathic membranous nephropathy]. Nihon Jinzo Gakkai Shi. (2007) 49:499–504. 17695812

[82] Tamano M Fuke Y Endo M Ohsawa I Fujita T Ohi H. Urinary complement factor H in renal disease. Nephron. (2002) 92:705–7. 10.1159/000064090 12372960

[83] Zipfel PF Heinen S Józsi M Skerka C. Complement and diseases: defective alternative pathway control results in kidney and eye diseases. Mol Immunol. (2006) 43:97–106. 10.1016/j.molimm.2005.06.015 16026839

[84] Kolev M Le Friec G Kemper C. Complement–tapping into new sites and effector systems. Nat Rev Immunol. (2014) 14:811–20. 10.1038/nri3761 25394942

[85] Arbore G Kemper C Kolev M. Intracellular complement - the complosome - in immune cell regulation. Mol Immunol. (2017) 89:2–9. 10.1016/j.molimm.2017.05.012 28601357 PMC7112704

[86] Guo J Ananthakrishnan R Qu W Lu Y Reiniger N Zeng S. RAGE mediates podocyte injury in adriamycin-induced glomerulosclerosis. J Am Soc Nephrol. (2008) 19:961–72. 10.1681/ASN.2007101109 18256352 PMC2386730

[87] Fu R Guo C Wang S Huang Y Jin O Hu H. Podocyte activation of NLRP3 inflammasomes contributes to the development of proteinuria in lupus nephritis. Arthritis Rheumatol. (2017) 69:1636–46. 10.1002/art.40155 28544564 PMC5568813

