# Supplementary figures and images for: Podocytes Produce and Secrete Functional Complement C3 and Complement Factor H
Source: Front Immunol. 2020 Aug 14;11:1833. doi: 10.3389/fimmu.2020.01833 (PMC7457071; doi:10.3389/fimmu.2020.01833)

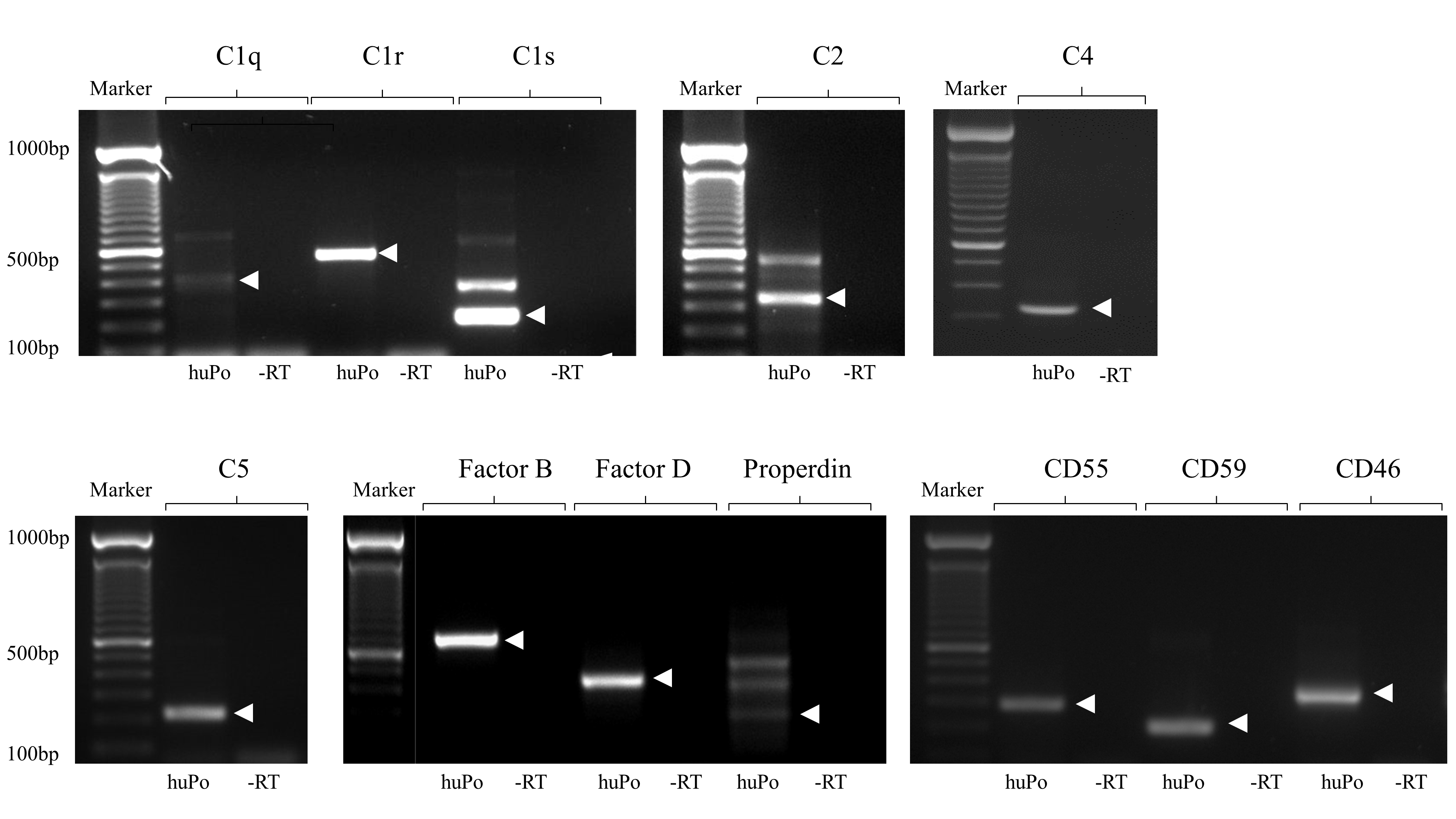

Supplement: Supplementary Figure 1 — Unstimulated human podocytes (HPC) express mRNA for various complement factors in conventional PCR (-RT, control of cDNA without addition of RT; bp, base pairs). [file Image_1.TIF]

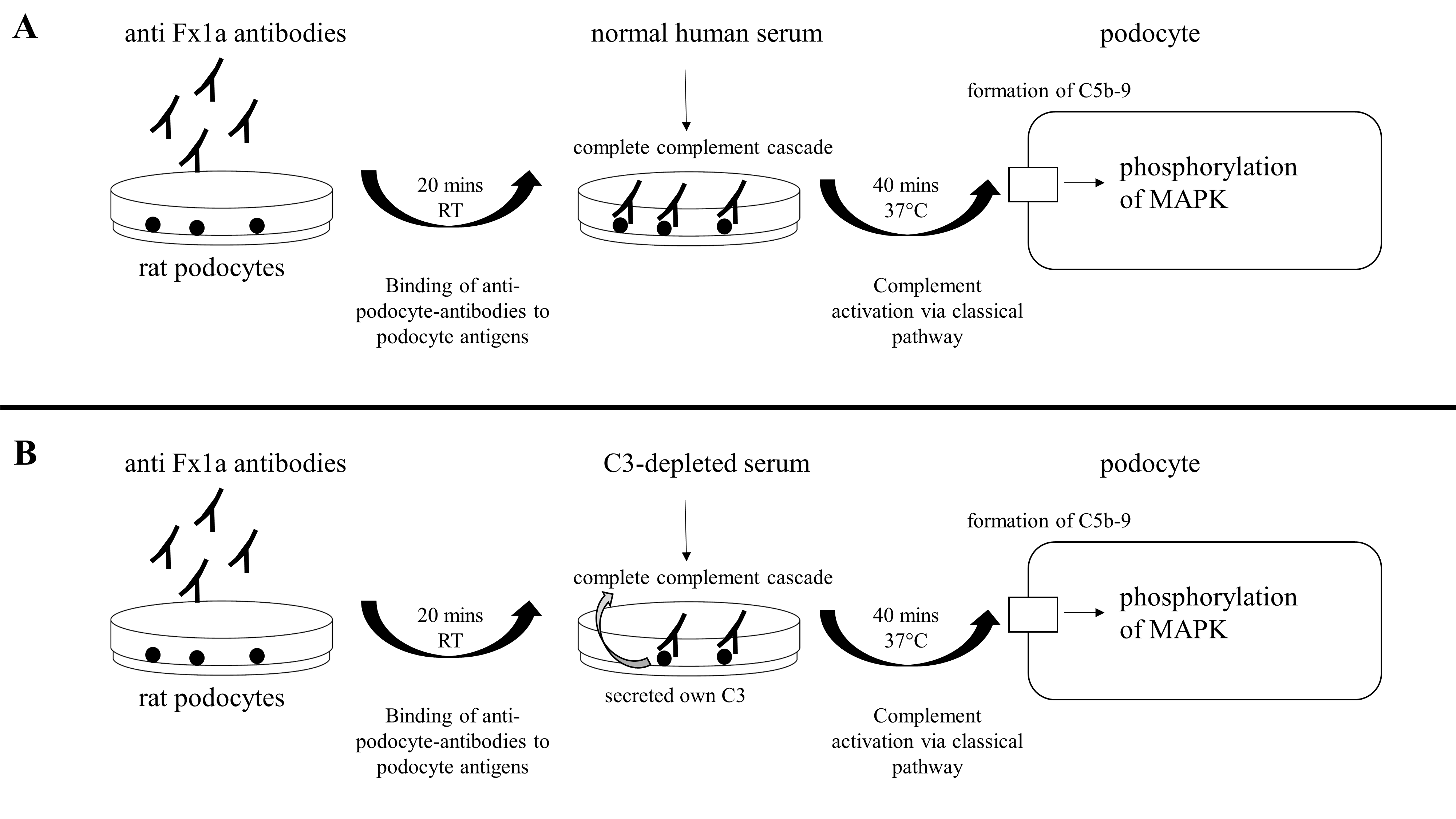

Supplement: Supplementary Figure 2 — Schematic presentation of the assumed underlying mechanisms in the complement challenge assay. (A) The binding of anti-podocyte antibodies leads to the activation of the complement cascade via the classical pathway (after the addition of normal human serum as a complete source of complement factors). The complement activation and building of C5b-9 induces sub-lytic damage in this in vitro model, which can be shown with the detection of phosphorylation of mitogen activated psrotein kinases (MAPK). (B) The use of C3-depleted serum did not prevent the phosphorylation of MAPK, which proves that podocytes derived C3 replaces external C3. [file Image_2.TIF]

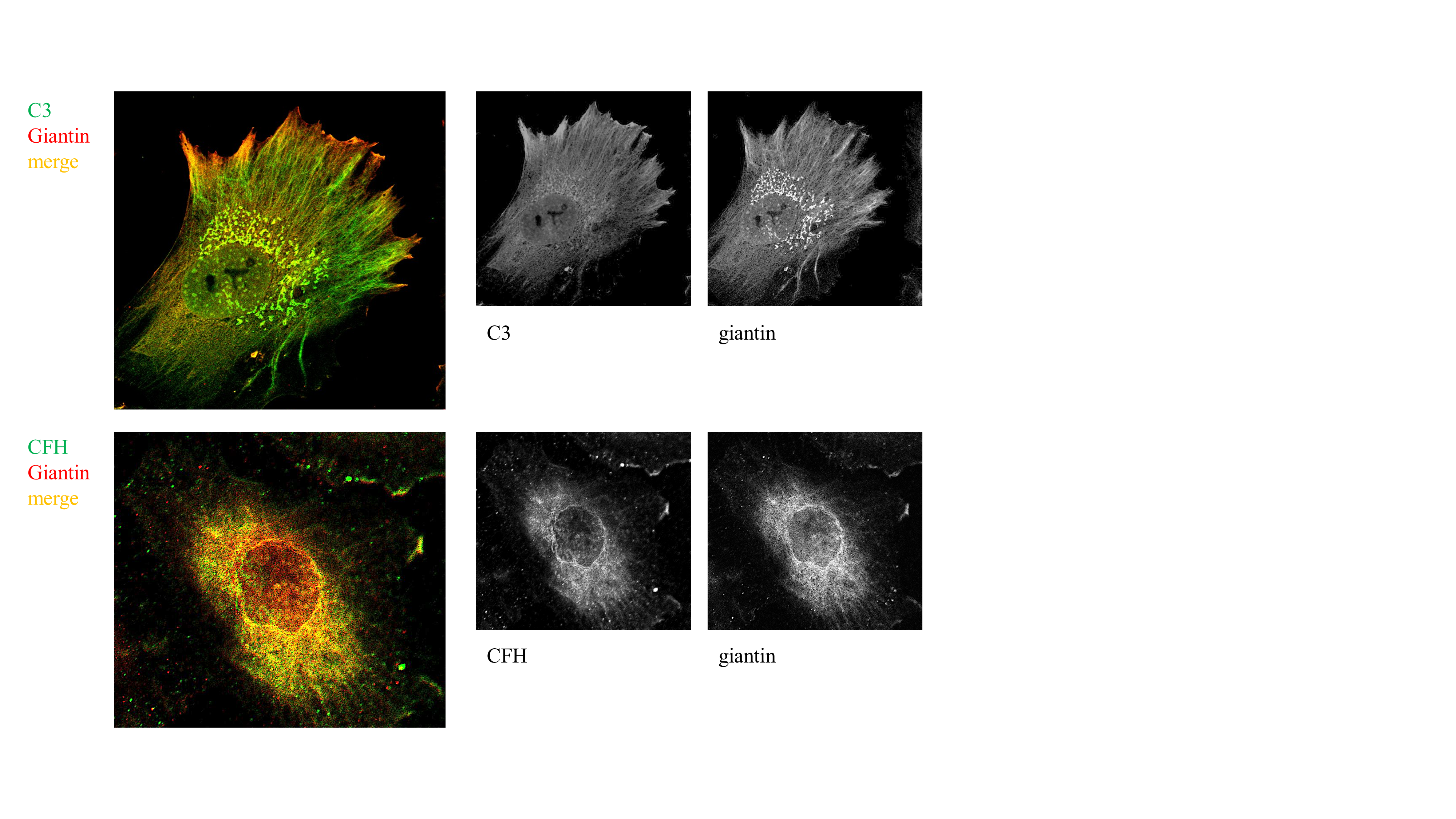

Supplement: Supplementary Figure 3 — Detailed spilt images: Podocytes were stained for C3 and CFH (green) and with an antibody against giantin, a Golgi apparatus protein (red). Co-localization is shown in yellow for both proteins (40x, scale bar 25 μm). [file Image_3.TIF]
