# Supplementary material for: Podocytes Produce and Secrete Functional Complement C3 and Complement Factor H
Source: Front Immunol. 2020 Aug 14;11:1833. doi: 10.3389/fimmu.2020.01833 (PMC7457071; doi:10.3389/fimmu.2020.01833)
Supplement: Supplementary Table 1 — Primer sequences conventional PCR, primers were obtained from Sigma Aldrich. [file Table_1.DOC]

**Supplementary Table 1**:

Primer sequences conventional PCR, primers were obtained from Sigma Aldrich:

| C1q forward | CAGGGATAAAAGGAGAGAAAGG |
| --- | --- |
| C1q reverse | TGGCGTGGTAGGTGAAGTAGTA |
| C1r forward | GATCTATGCCAACGGGAAGA |
| C1r reverse | CATTCTTCCAAATGCCCTGT |
| C1s forward | AAGAGCGTTTTACGGGGTTT |
| C1s reverse | AATCTCCCCAATCAGTGCAG |
| C2 forward | CCTTGAATGGGAGCAAACTGAAC |
| C2 reverse | GATTGATGTGAAAGTCTCGTGGC |
| C5 forward | AGTGTGTGGAAGGGTGGAAG |
| C5 reverse | GTTCTCTCGGGCTTCAACAG |
| factor B forward | GTG TGA CCA CCA CTC CAT GG |
| factor B reverse | CCA TCC TCA GCA TCG ACT CC |
| factor D forward | CGACCACGACCTCCTGCTGCTACA |
| factor D reverse | GCTCGGGACTTTGTTGCTTGGGTG |
| properdin forward | GATGGGCGGCTGGTCTGGCTG |
| properdin reverse | TGCGGCTTCGTGTCTCCTTAG |
